# Multiparametric and accurate functional analysis of genetic sequence variants using CRISPR-Select

**DOI:** 10.1038/s41588-022-01224-7

**Published:** 2022-12-05

**Authors:** Yiyuan Niu, Catarina A. Ferreira Azevedo, Xin Li, Elahe Kamali, Ole Haagen Nielsen, Claus Storgaard Sørensen, Morten Frödin

**Affiliations:** 1 Biotech Research and Innovation Centre (BRIC), Faculty of Health Sciences, University of Copenhagen, Copenhagen, Denmark; 2 Department of Gastroenterology, Herlev Hospital, University of Copenhagen, Copenhagen, Denmark

**Keywords:** Mutagenesis, Cancer prevention, Genetics research, Genetic testing, High-throughput screening

## Abstract

Determining the functional role of thousands of genetic sequence variants (mutations) associated with genetic diseases is a major challenge. Here we present clustered regularly interspaced short palindromic repeat (CRISPR)-Select^TIME^, CRISPR-Select^SPACE^ and CRISPR-Select^STATE^, a set of flexible knock-in assays that introduce a genetic variant in a cell population and track its absolute frequencies relative to an internal, neutral control mutation as a function of time, space or a cell state measurable by flow cytometry. Phenotypically, CRISPR-Select can thereby determine, for example, pathogenicity, drug responsiveness/resistance or in vivo tumor promotion by a specific variant. Mechanistically, CRISPR-Select can dissect how the variant elicits the phenotype by causally linking the variant to motility/invasiveness or any cell state or biochemical process with a flow cytometry marker. The method is applicable to organoids, nontransformed or cancer cell lines. It is accurate, quantitative, fast and simple and works in single-well or 96-well higher throughput format. CRISPR-Select provides a versatile functional variant assay for research, diagnostics and drug development for genetic disorders.

## Abstract (web summary)

CRISPR-Select is a quantitative assay for the functional impact of genetic variants, including pathogenicity, drug response, oncogenicity, cell motility and other cell states.

## Main

Myriads of genetic sequence variants are being revealed by next-generation sequencing (NGS) across diseases with a genetic origin (https://www.ncbi.nlm.nih.gov/clinvar/)^1^. Unfortunately, the largest class of variants are variants of uncertain significance (VUS), as opposed to variants of known benign or pathogenic role. VUS account for >41% of all identified variants, probably much more, as VUS findings are often not reported (https://clinvarminer.genetics.utah.edu)^2,3^. The causative genes for the 5,000–8,000 human monogenic diseases have produced VUS by the hundreds of thousands and cancer VUS amount to millions^1^. For the hereditary breast and ovarian cancer genes, *BRCA1* and *BRCA2*, for example, 68,962 variants are reported as VUS and only 6,258 as benign or pathogenic (https://brcaexchange.org/; November 2022)^1,4^. VUS are mainly missense, putative splice-site and small in-frame insertion or deletion (InDel) mutations for which functional consequences are difficult to predict. VUS represent a huge medical problem by precluding molecular diagnosis, risk prediction, patient counseling and treatment, such as prophylactic surgery or targeted therapy. VUS also impede our understanding of the basic mechanisms of genetic diseases.

Functional genetic assays have the capacity to classify VUS as benign or pathogenic and predict drug response and, therefore, are increasingly in demand in the clinical genetics community^3,5,6^. Functional genetic assays are equally important research tools to answer fundamental biological questions as, for example, how does a specific sequence variant impact cell phenotype and what is the mechanism? Finally, functional genetic assays may facilitate development of targeted therapies, providing isogenic cell screening systems, patient stratification and companion diagnostics. So far, however, the vast majority of disease genes lack tailored functional assays that are reliable, flexible, cost-effective and sufficiently fast for use in research, in the clinic, or in drug development.

Genome editing technologies, such as clustered regularly interspaced short palindromic repeats (CRISPR), potentially can provide gold-standard functional assays, as they allow analysis of variants in their proper genomic and cellular context^7–11^. This was illustrated by recent large-scale variant screens that employed DNA templated knock-in of variants^12^ or base editing^13,14^. These approaches were performed in a multiplexed format: a library of variants assayed in a cell population, allowing results within few weeks and avoiding issues with clonal variation. Screen hits, however, may need validation experiments, loss-of-function variants generally manifested only with haploinsufficient genes or when screening in a haploid cell line, results were not quantitative and absolute variant frequencies with derived controls were not obtained. Base editing screens, moreover, provide hits for a small fraction of the tested variants only. Finally, the screening readouts were limited to cell proliferation and/or survival, precluding analysis of variants for the majority of the 5,000–8,000 monogenic diseases^15^ that do not involve abnormal proliferation/survival.

Conventional CRISPR variant analysis based on the generation and analysis of clonal cell lines harboring the variant can validate screen hits and allows readouts beyond proliferation/survival. Clonal analysis, however, is time-consuming and laborious, suffers from clonal variation artifacts and precludes studies on variants that block cell proliferation or survival.

We, therefore, developed a flexible set of functional assays for variant evaluation that accommodate the strengths of the previous approaches while eliminating shortcomings: CRISPR-Select^TIME^, CRISPR-Select^SPACE^ and CRISPR-Select^STATE^. These cell population-based knock-in assays are arrayed (one variant per cell population) and all track absolute variant frequencies in the cell population relative to a synonymous (that is, neutral) normalization mutation, but in different ways that enable the following distinct readouts: CRISPR-Select^TIME^ tracks variant frequencies as a function of time to determine effects on cell proliferation and/or survival. CRISPR-Select^SPACE^ tracks variant frequencies in the spatial dimension to assay effects on, for example, cell migration or invasiveness. CRISPR-Select^STATE^ tracks variant frequencies as a function of a fluorescence-activated cell sorting (FACS) marker level and can thereby determine variant effects on essentially any physiological/pathological state or biochemical process of a cell.

Altogether, CRISPR-Select can determine variant effects on essentially any cell parameter and in any cell type. The method is fast, quantitative and scalable. It is highly reliable because the assay controls for sufficient cell numbers underlying the data, clonal variation, CRISPR off-target effects, false negatives and other experimental confounders.

## Results

### CRISPR-Select is a multiparametric functional variant assay

**Fig. 1:**
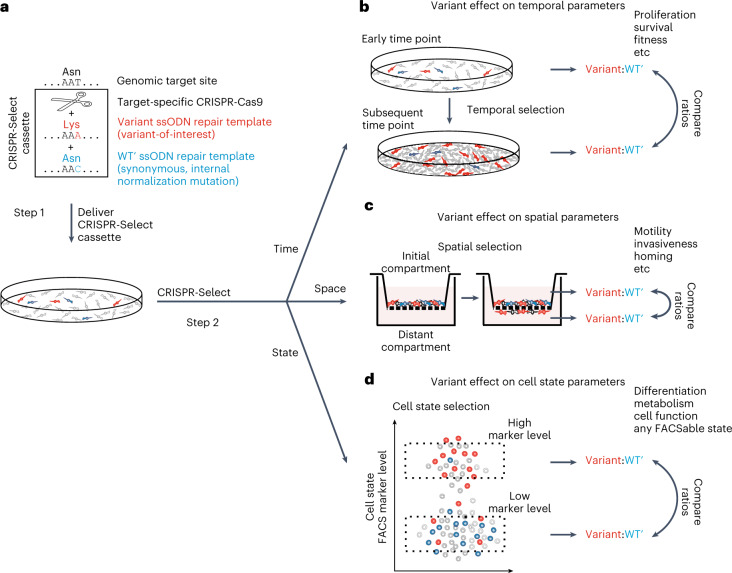
Principle of CRISPR-Select multiparametric and accurate functional analysis of genetic sequence variants. **a**, In step 1, a cell population of interest is transfected with a CRISPR-Select cassette composed of target-specific CRISPR-Cas9 and two ssODN repair templates that are identical, except that one harbors the variant of interest and the other a synonymous, internal normalization mutation (WT′). In step 2, the difference in the ratios of cells with knock-in of the variant relative to WT′ is determined as a function of either a temporal parameter (CRISPR-Select^TIME^), a spatial parameter (CRISPR-Select^SPACE^) or a cell state parameter (CRISPR-Select^STATE^). **b**, For CRISPR-Select^TIME^, comparison of variant:WT′ ratios at an early and a subsequent time point determines selection for or against the variant, which is a readout of variant effect on cell proliferation, survival or fitness. **c**, For CRISPR-Select^SPACE^, comparison of variant:WT′ ratios in an initial compartment and a spatially distant compartment determines the selective effect of the variant on cell motile/invasive/homing or similar properties. **d**, For CRISPR-Select^STATE^, comparison of variant:WT′ ratios in two cell populations FACS isolated according to different levels of a marker for any cell-state-of-interest determines the effect of the variant on that cell state.

We developed a well-controlled functional genetic assay, which is based on a CRISPR-Select cassette comprising the following: (1) a CRISPR-Cas9 reagent designed to elicit a DNA double-strand break close to the genomic site to be mutated, (2) a single-stranded oligodeoxynucleotide (ssODN) repair template containing the variant of interest to be knocked in and (3) a second ssODN repair template with a synonymous, internal normalization mutation termed WT prime (WT′) at the same, or nearly the same position as the variant of interest and otherwise identical to the first ssODN (Fig. 1a). The guide (g)RNA used is chosen such that the variant and WT′ mutations are located in the seed region or protospacer-adjacent motif (PAM) of the CRISPR-Cas9 binding site to minimize postknock-in recutting. In step 1, the CRISPR-Select cassette is delivered to a cell population of interest.

In step 2, differences in the ratio of cells with knock-in of variant relative to WT′ are measured as paired determinations in aliquots of the cell population as a function of a temporal parameter (CRISPR-Select^TIME^), whereby the functional readouts are cell proliferation and survival (Fig. 1b), a spatial parameter (CRISPR-Select^SPACE^), producing functional readouts of cell motility, invasiveness or similar properties (Fig. 1c), or a cell state parameter measurable by FACS (CRISPR-Select^STATE^), which allows functional readouts of any physiological or pathological cell state or cell process with a FACS marker (Fig. 1d).

**Fig. 2:**
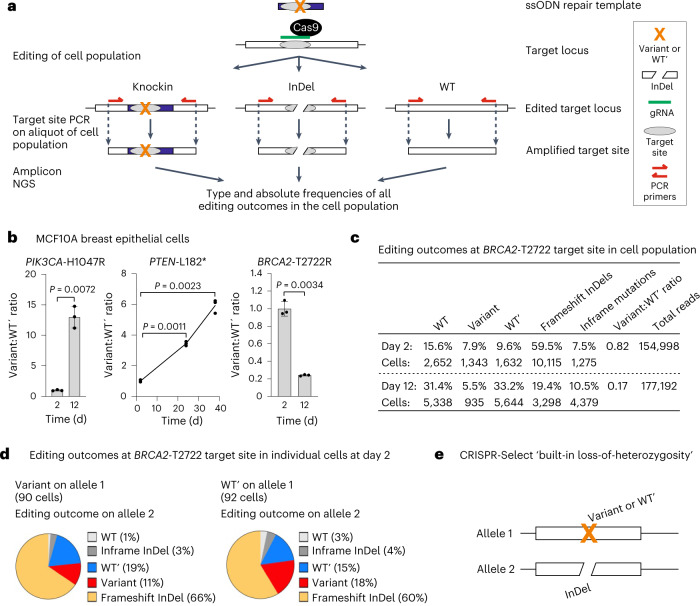
CRISPR-Select^TIME^ proof-of-concept with analysis of oncogene and tumor suppressor variants. **a**, Schematic of the CRISPR-Select target site PCR and amplicon NGS determination of variant and WT′ frequencies in the edited cell population. The schematic highlights that the method samples and determines the types and absolute frequencies of all editing outcomes (alleles) in the cell population, because PCR primers anneal to unmodified sequences outside the region covered by the ssODNs. **b**, CRISPR-Select^TIME^ analysis of selection effects of known cancer driver variants in *PIK3CA*, *PTEN* or *BRCA2*. Cassettes for the variants were delivered to iCas9-MCF10A cells, and variant:WT′ ratios were determined on day 2 and at indicated later time points and normalized to day 2 value. Data are means ± s.d. of *n* = 3 independent biological replicates. *P* values are from two-tailed paired *t*-tests. **c**, Editing outcomes at the *BRCA2-*T2722 target site in one of the *BRCA2* assay replicates in (**b**), as determined by amplicon NGS on the cell population. Based on the edit frequencies and the known genomic DNA input for the NGS analysis, the approximate number of cells that harbor the various edits can be calculated. **d**, Editing outcomes at the *BRCA2-*T2722 target site on both alleles in individual cells from one of the *BRCA2* assay replicates in (**b**), as determined by amplicon sanger sequencing on single cells. **e**, Schematic illustrating the ‘built-in loss of heterozygosity’ feature inherent to CRISPR-Select: a majority of cells with variant or WT′ on one allele will have the other allele inactivated by a disruptive editing outcome such an InDel.

As a key feature, CRISPR-Select can control that variant:WT′ ratios are based on the sufficient numbers of knock-in cells for accurate determination of variant effects: Editing outcomes are quantitated by genomic PCR amplification of the target site on an aliquot of the cell population with primers annealing to sequences outside the region covered by the ssODNs, followed by amplicon NGS (Fig. 2a). CRISPR-Select thereby determines the types and frequencies of all editing outcomes in the cell population. Based on the known genomic template amounts for the PCR, absolute numbers of knock-in alleles, which approximate knock-in cells, can be calculated.

We first tested CRISPR-Select^TIME^ with known driver mutations in breast cancer and therefore probed their effects in the patient-relevant MCF10A model, which are immortalized, but otherwise normal and diploid, human breast epithelial cells. The cells were engineered for doxycycline-inducible expression of Cas9 (iCas9-MCF10A), such that the CRISPR-Select cassette was delivered by doxycycline pretreatment to induce Cas9 and lipofection of synthetic gRNA and ssODNs. First, we analyzed the most frequent gain-of-function mutation H1047R in the proto-oncogene *PIK3CA*^16^. As PIK3CA (phosphatidylinositol-3-kinase) mediates growth factor receptor signaling for proliferation and survival, the experiment was performed under serum- and growth factor-depleted culture conditions. Indeed, CRISPR-Select^TIME^ detected a ~13-fold enrichment of *PIK3CA-*H1047R variant cells over time, consistent with the known driver function of the variant (Fig. 2b).

In a similar experiment, we assessed the known loss-of-function mutation L182* (ref. ^17^) in the tumor suppressor gene *PTEN*, encoding the major negative regulator of PIK3CA. CRISPR-Select^TIME^ determined accumulation of cells with *PTEN*-L182*, in accordance with the established driver function of this variant (Fig. 2b).

Finally, we tested the expert panel assessed pathogenic (loss-of-function) T2722R variant in the tumor suppressor gene *BRCA2* (ref. ^18^), encoding a key factor for homologous recombination DNA repair, essential for proliferating cells^19^. Accordingly, CRISPR-Select^TIME^ revealed a ~5-fold loss of cells with the *BRCA2-*T2722R variant over time (Fig. 2b). In conclusion, CRISPR-Select^TIME^ can reveal gain-of-function mutations in oncogenes and loss-of-function mutations in tumor suppressor genes.

We used *BRCA2-*T2722R (Fig. 2b) to illustrate that CRISPR-Select can control for sufficient numbers of knock-in cells underlying the results as follows: with 100 ng genomic DNA as PCR template (~17,000 diploid cells) and knock-in frequencies for T2722R and WT′ of 8–9%, as determined by NGS, it can be calculated that the experiment tracked the fate of approximately 1,300–1,600 T2722R or WT′ cells from day 2 and onwards (Fig. 2c). With such a large population of knock-in cells, confounding effects from potential clonal variation are also effectively diluted out.

**Extended Data Fig. 1:**
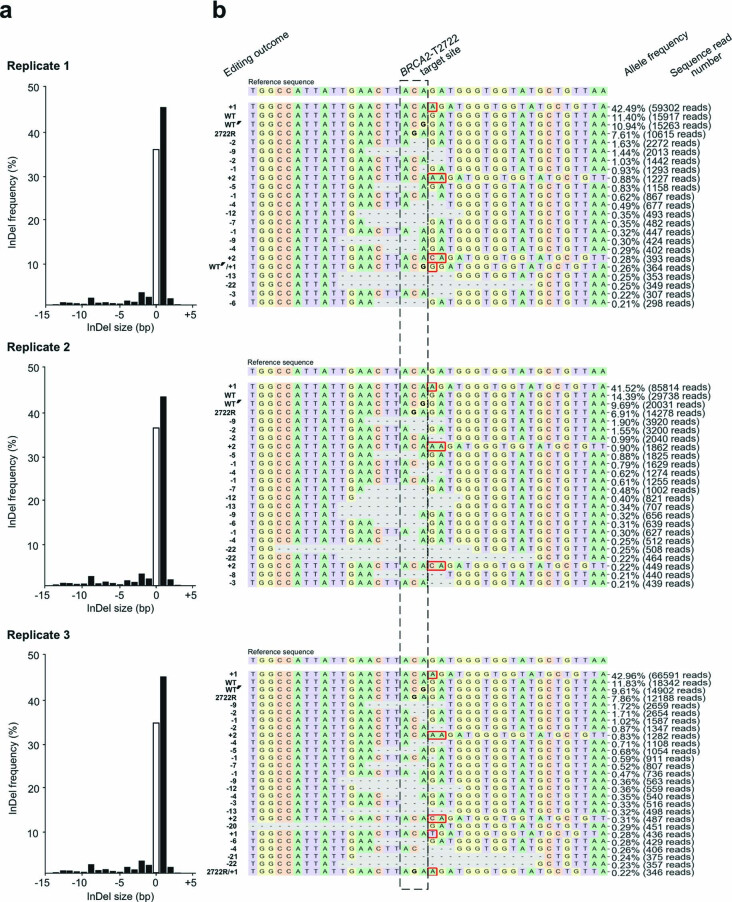
CRISPR-Select editing outcomes at the *BRCA2*-T2722 target site at the cell population level in three replicate experiments. A cassette for *BRCA2-*T2722R mutation was delivered to iCas9-MCF10A cells. On day 2, editing outcomes at the BRCA2-*T2722* target site were determined by PCR amplification of the target site on genomic DNA from an aliquot of the edited cell population, followed by NGS of the amplicons. **a**, Bar graph representation of frequencies of the various detected InDels, shown as percent of all sequence reads in the sample. Insertions and deletions are indicated by (+) and (-), respectively. **b**, Sequences of detected alleles with frequency >0.20%. The nature of the editing outcome is indicated in the column preceding the sequences and insertions and deletions are indicated by (+) and (-), respectively. Within the sequences, ssODN-specified mutations are given in boldface. Insertions and deletions are indicated by red boxes and stippled lines, respectively. The frequency as percent and the number of sequence reads for each allele are shown following the sequences. Note the nearly identical InDel profiles for the three replicates. This stems from the highly reproducible repair by NHEJ and MMEJ pathways, dictated by the specific sequence surrounding the DNA double-strand break (reviewed in^47^). This reproducible repair ensures reproducible built-in loss-of-heterozygozity in CRISPR-Select assays in diploid cells.

CRISPR-Select showed large effect sizes with variants in the recessive tumor suppressors *BRCA2* and *PTEN*^20^ in diploid MCF10A cells (Fig. 2b). This suggests that most cells with variant knock-in on one allele had obtained an inactivating editing outcome on the other allele, which was supported by the NGS analysis. For example, frameshift (that is, knock-out) InDels were ~5-fold more frequent than variant knock-in events in the *BRCA2-*T2722R edited cell population (Fig. 2c and Extended Data Fig. 1), in accordance with the notion that InDel repair is much more efficient than knock-in repair^21^.

**Extended Data Fig. 2:**
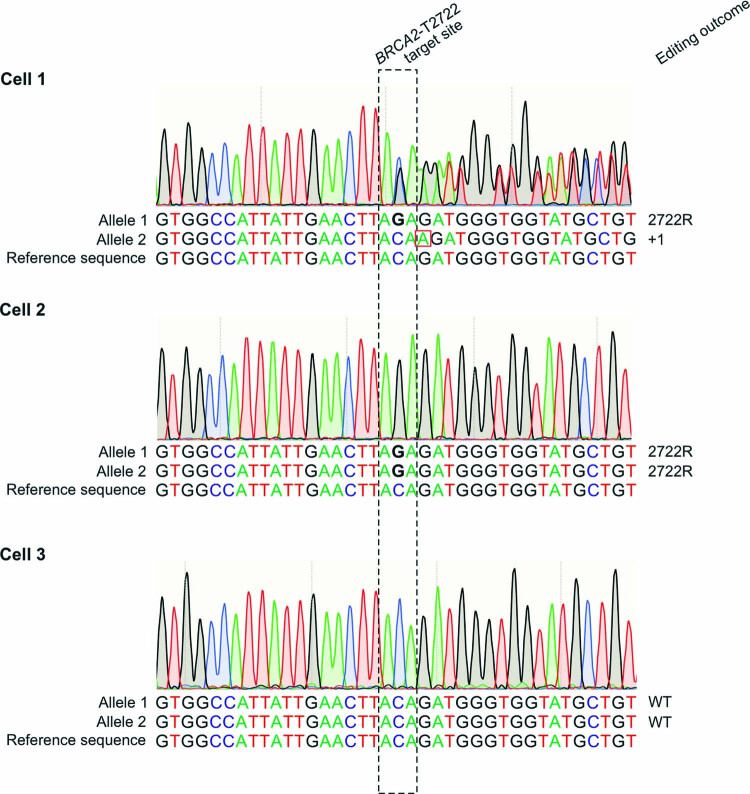
CRISPR-Select editing outcomes at the *BRCA2*-T2722 target site in three individual cells. A cassette for *BRCA2-*T2722R mutation was delivered to iCas9-MCF10A cells. On day 2, individual cells were isolated by FACS and editing outcomes at the *BRCA2-T2722* target site were determined by PCR amplification of the target site on genomic DNA from the individual cells, followed by Sanger sequencing of the amplicons. Results from three individual cells are shown. The nature of the editing outcome is indicated in the column following the sequences, with insertion indicated by (+). Within the sequences, ssODN repair template-specified mutations are given in boldface and insertion is indicated by a red box.

We demonstrated that this knock-in:InDel frequency pattern is also observed in individual cells by Sanger sequencing the *BRCA2-*T2722R target site PCR amplified from ~500 single cells FACS isolated on day 2 (examples shown in Extended Data Fig. 2). Of the 90 cells having T2722R knock-in on one allele, 66% had a frameshift InDel on the other allele (Fig. 2d), mirroring the knock-in:InDel pattern from the cell population data. Furthermore, 11% of the cells with T2722R knock-in on one allele had the same mutation on the other allele and 3% had an in-frame InDel, and as evident from the NGS data, virtually all in-frame InDels destroyed T2722 (Extended Data Fig. 1d). Thereby, these latter scenarios also created overall BRCA2 loss. Of the 92 cells with WT′ on one allele, a very similar distribution of editing outcomes on the other allele was observed (Fig. 2d).

These data support two conclusions. First, the two cell populations that are compared in the CRISPR-Select analysis, that is, cells having either variant of interest or WT′ on one allele, have the same type of editing heterogeneity on the other allele at the early time point. Therefore, any difference (loss or gain) in frequency of variant compared to WT′ cells at subsequent time points can be conclusively ascribed to an effect of the variant. Second, CRISPR-Select functions such as to have ‘built-in loss of heterozygosity’ (Fig. 2e): A majority of cells with knock-in of variant of interest on one allele will also have the other allele inactivated by a disruptive editing outcome.

**Extended Data Fig. 3:**
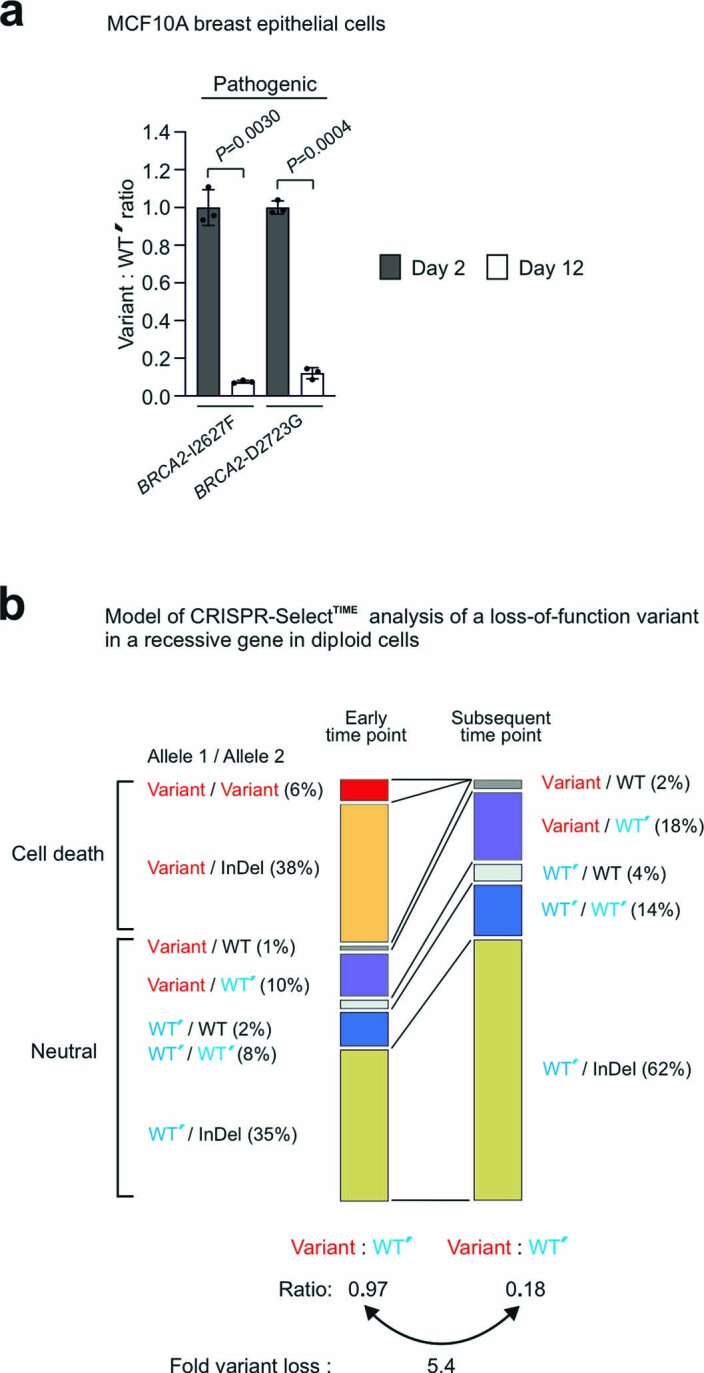
CRISPR-Select^TIME^ detects loss-of-function variants in recessive, essential genes in diploid cells. **a**, Cassettes for known pathogenic (loss-of-function) *BRCA2* variants were delivered to diploid iCas9-MCF10A cells and variant:WT’ ratios were determined at time points, as indicated. Variant:WT′ ratios were normalized to the day 2 value. Data are means+/− s.d. of *n* = 3 independent biological replicates. P values are from two-tailed paired t-tests. **b**, Schematic illustration highlighting the various combinations of editing outcomes on the two alleles in diploid cells, the predicted cellular effect and variant:WT′ ratios at early and subsequent time points, when analysing a loss-of-function variant in a recessive, essential gene by CRISPR-Select^TIME^. The illustration is a model, but uses early time point data that approximate the day 2 data of the *BRCA2-*T2722R single-cell analysis (Fig. 2d). The model assumes that the variant and InDels are profound loss-of-function events. With this assumption and the editing outcomes shown, variant alleles can be expected to exhibit a 5-fold loss relative to WT′ from early to subsequent time point, which agrees well with the experimental analysis of *BRCA2-*T2722R (Fig. 2b). Loss-of-function variants in haploinsuficient genes can be expected to behave in a similar manner, except that variant loss will be more pronounced due to the haploinsufficiency.

Combined, these features explain why CRISPR-Select works in normal diploid cells to robustly reveal the effect of a variant, including loss-of-function variants in recessive genes. We confirmed this notion with two additional expert panel-assessed loss-of-function *BRCA2* missense variants in diploid MCF10A cells (Extended Data Fig. 3a). A schematic of allelic editing combinations and predicted CRISPR-Select result for loss-of-function variants in recessive genes in diploid cells is shown in Extended Data Fig. 3b.

### CRISPR-Select^TIME^ molecular diagnosis and drug response testing

**Extended Data Fig. 4:**
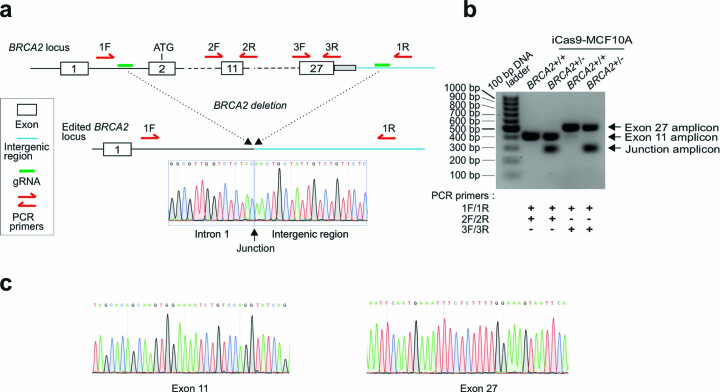
Generation of iCas9-MCF10A-*BRCA2*+/− cells. **a**, Outline of strategy for deletion of all *BRCA2* coding exons, using CRISPR-Cas9 editing with dual gRNAs, indicated by green bars in the schematic of the wild-type *BRCA2* locus. The location of PCR primers for genotyping of expanded clones is also indicated. The lower part of the panel shows the edited *BRCA2* locus, as well as a chromatogram from Sanger sequencing of PCR products that have amplified the junction sequence after *BRCA2* deletion. **b**, Representative PCR genotyping results obtained for all genotyped clones, showing agarose gel separated amplicons that identify iCas9-MCF10A expanded clones as either *BRCA2*+/+ or *BRCA2*+/−. **c**, Chromatograms from Sanger sequencing of PCR products that have amplified exon 11 or exon 27 from wild-type *BRCA2* alleles in *BRCA2*+/− clones.

Given the BRCA2 results, we explored further, whether CRISPR-Select may be used for molecular diagnosis of hereditary breast and ovarian cancer. While the ‘built-in loss of heterozygosity’ of CRISPR-Select may suffice for research purposes, diagnostic use requires a defined genetic setting. We, therefore, generated iCas9-MCF10A-*BRCA2*^+/−^ cells with all *BRCA2* coding exons on one allele deleted (Extended Data Fig. 4).

**Fig. 3:**
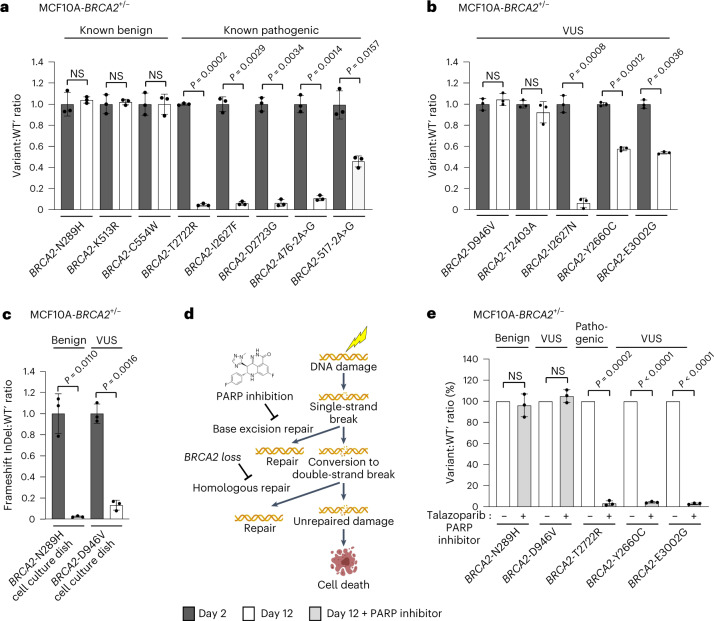
CRISPR-Select^TIME^ classification of *BRCA2* variants and prediction of PARP inhibitor response. Cassettes for various *BRCA2* variants were delivered to iCas9-MCF10A-*BRCA2*^+/−^ cells and variant:WT′ ratios were determined at time points, as indicated. **a**, Correct classification of known benign and pathogenic *BRCA2* variants as being neutral and negatively selected for, respectively. **b**, Selection effect of *BRCA2* VUS. **c**, Internal frameshift InDel control for neutral variants. Frameshift InDel:WT′ ratios were determined in the samples from the cell culture dishes for CRISPR-Select analysis of *BRCA2-*N289H in (**a)** or *BRCA2-*D946V in (**b)**. **d**, Schematic illustrating that blockade of base excision repair by PARP inhibition and homologous repair by *BRCA2* loss can cause synthetic lethality after DNA damage. **e**, Correct classification of *BRCA2* variants as being resistant or sensitive to PARP inhibition. On day 2 after delivery of cassettes for neutral (N289H and D946V) or loss-of-function (T2722R, Y2660C and E3002G) *BRCA2* variants, cells were split and cultured from days 3–12 in the presence of vehicle (−) or talazoparib (+). All variant:WT′ or frameshift:WT′ ratios were normalized to the day 2 value, except for (**e**), where ratios on day 12 were normalized to the values obtained with vehicle set to 100%. Data are mean ± s.d. of *n* = 3 independent biological replicates. *P* values are from two-tailed paired *t*-tests. NS, not significant (*P* > 0.05).

**Extended Data Fig. 5:**
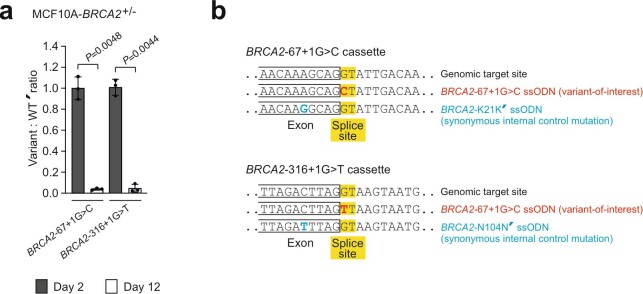
CRISPR-Select^TIME^ analysis of non-coding variants in mRNA splice sites. **a**, Cassettes for known pathogenic (loss-of-function) *BRCA2* variants affecting intronic splice site sequences were delivered to iCas9-MCF10A-*BRCA2*+/− cells and variant:WT′ ratios were determined at time points, as indicated. Variant:WT′ ratios were normalized to the day 2 value. Data are means+/− s.d. of *n* = 3 independent biological replicates. P values are from two-tailed paired t-tests. **b**, In cassettes for intronic splice site variants, WT′ was placed slightly off-set of variant and into the exon, so as not to be located in the splice site, where it would not be neutral.

We first tested expert panel-assessed benign or pathogenic *BRCA2* variants, the latter including the missense variants tested in diploid MCF10 cells, as well as intronic splice site variants. In accordance with the expected result, CRISPR-Select^TIME^ detected no effect of the benign variants and a partial or complete (~20-fold) cell loss for the pathogenic variants on day 12 after transfection (Fig. 3a and Extended Data Fig. 5a). When employing cassettes for splice site variants, we placed WT′ slightly off-set of variant and into the exon to avoid location within the splice site (Extended Data Fig. 5b). When testing five *BRCA2* VUS from ClinVar, two were neutral, whereas three evoked complete or partial cell loss (Fig. 3b). The apparently benign/neutral variants could be false negatives due to lack of selection pressure for various reasons in the particular experiments. However, the complete NGS characterization of editing outcomes by CRISPR-Select allows analysis of frameshift InDel:WT′ ratios, which demonstrated strong negative selection against cells with frameshift InDels in the same cell culture dishes, where the benign/neutral *BRCA2-*N289H and *BRCA2-*D946V variants were not selected against (Fig. 3c). CRISPR-Select thereby has built-in, internal controls showing that apparently neutral variants are truly neutral.

Inhibitors of poly(ADP-ribose) polymerase 1 (PARP1) are used as synthetic lethal therapies for tumors with BRCA loss of function (Fig. 3d)^22,23^. We tested whether CRISPR-Select can predict patient response to PARP inhibition by culturing various *BRCA2* variants in the absence or presence of talazoparib, a PARP inhibitor. Intriguingly, CRISPR-Select^TIME^ correctly grouped cells with neutral *BRCA2* variants as insensitive to PARP inhibition, whereas loss-of-function *BRCA2* variants dramatically sensitized cells to PARP inhibitor killing beyond the effect of *BRCA2* loss itself, normalized to 100% (Fig. 3e).

**Extended Data Fig. 6:**
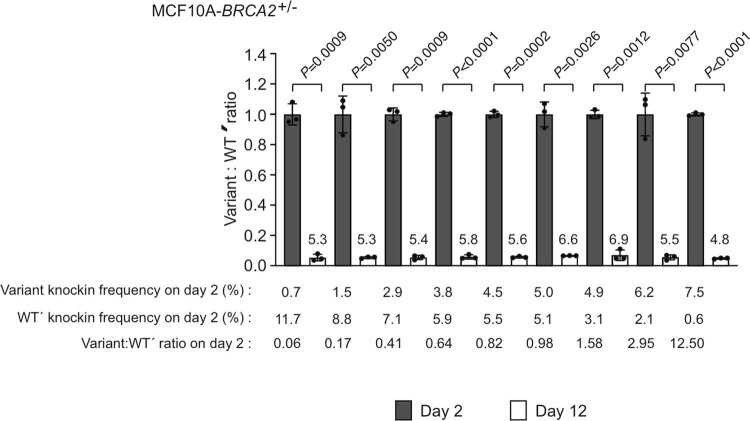
CRISPR-Select^TIME^ quantitation of variant effects is independent of variant:WT′ knockin ratios at the early time point (day 2). CRISPR-Select cassettes with ssODNs for the pathogenic *BRCA2-*T2722R variant and WT′ mixed at various stoichiometries were delivered to iCas9-MCF10A-*BRCA2*+/− cells, eliciting knockin of variant and WT′ over a wide range of ratios on day 2, as indicated below the x-axis. On the y-axis, bars show variant:WT′ ratios normalized to the day 2 value. The numbers above the day 12 bar show the value in % of the day 2 value. Data are means+/− s.d. of *n* = 3 replicate transfections. P values are from two-tailed paired t-tests.

Some variant:WT′ pairs were not knocked in at a ratio of ~1 at day 2, but at lower (0.4; *BRCA2-*Y2660C) or higher (2.5; I2627N) ratios. However, CRISPR-Select determined the same effect size of a variant at day 12 for any day 2 variant:WT′ ratio between 0.06 and 12.5, which we demonstrated by delivering variant and WT′ ssODNs at a wide range of stoichiometries (Extended Data Fig. 6). CRISPR-Select robustly determines variant effects despite of skewed initial variant:WT′ ratios, because it is based on measurement of the *relative change* in variant:WT′ ratios.

### CRISPR-Select^TIME^ drug target, resistance and on-target assays

**Fig. 4:**
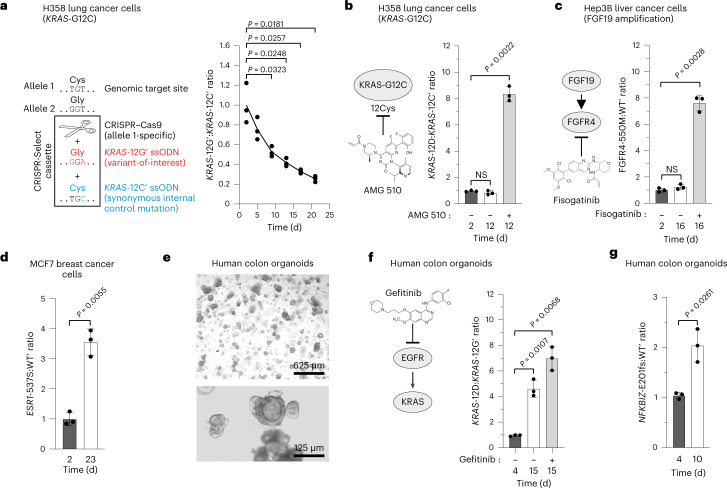
CRISPR-Select^TIME^ identification of drug targets, drug resistance/on-target actions and disease mechanisms in cancer cells and organoids. **a**, Allele-specific correction of KRAS-12C driver variant to proto-oncogenic KRAS-12G′ in H358 lung cancer cells causes negative selection. **b**, After allele-specific mutation of KRAS-12C to KRAS-12D in H358 cells, the cells were split on day 2 and cultured from days 3–12 in the presence of vehicle (−) or AMG 510 (+). **c**, After delivery of a cassette for V550M mutation in FGFR4 in Hep3B liver cancer cells, the cells were split on day 2 and cultured from days 3–16 in the presence of vehicle (−) or fisogatinib (+). **d**, Positive selection for the tamoxifen-resistant *ESR1*-Y537S variant in MCF7 breast cancer cells. **e**, Representative images of the human primary colon epithelial organoids used (low and high magnification), showing proper colonic organoid structures obtained in all performed experiments. **f**, After delivery of a cassette for KRAS-G12D mutation in colon epithelial organoids, the cells were split on day 4 and cultured from days 4–15 in the presence of vehicle (−) or gefitinib (+). **g**, Positive selection for ulcerative colitis *NFKBIZ*-E201fs variant in colon epithelial organoids cultured in the absence of Noggin. All variant:WT′ (or variant:variant′) ratios were normalized to the day 2 (or day 4 for organoids) value. Data are mean ± s.d. of *n* = 3 independent biological replicates. *P* values are from two-tailed paired *t*-tests. NS, not significant (*P* > 0.05).

We tested the potential of our method to identify cancer drivers and thereby candidate drug targets in human cancer cells, using H358 lung cancer cells with the recurrent mono-allelic KRAS-G12C driver mutation as an example^24^. We nucleofected H358 cells with a CRISPR-Select cassette in the form of ribonucleoprotein (RNP; Cas9 protein and synthetic gRNA) and repair ssODNs for allele-specific correction of KRAS-12C to KRAS-12G′ (that is, WT) or mutation to a synonymous KRAS-12C′ form (Fig. 4a). CRISPR-Select^TIME^ revealed a loss of KRAS-12G′-corrected cells over time, demonstrating KRAS-12C dependence for proliferation and/or survival. Accordingly, lung cancer with KRAS-12C is recently being targeted with AMG 510 (https://www.fda.gov/), which blocks the mutant through covalent binding to the 12C residue^24^.

Some patients treated with AMG 510 may anticipate recurrence of tumors harboring another oncogenic KRAS mutation, KRAS-12D, which is insensitive to AMG 510 (ref. ^24^). To test whether CRISPR-Select^TIME^ can model such resistance, we delivered a cassette for allele-specific mutation of KRAS-12C to KRAS-12D or to a synonymous KRAS-12C′ form. In the absence of AMG 510, KRAS-12D provided no selective advantage, but in the presence of AMG 510, cells with KRAS-12D accumulated ~8-fold compared to cells with KRAS-12C′ in the H358 cell population over time (Fig. 4b), demonstrating KRAS-12D mutation as an AMG 510 resistance mechanism. Of equally high importance, by such type of analysis, that is, mutagenesis of the drug-binding site and measuring that drug effect disappears, CRISPR-Select^TIME^ can determine that drugs act via the intended target to elicit their effect.

We demonstrated the generality of this notion using human Hep3B liver cancer cells with focal amplification of the growth factor gene FGF19, which represent liver cancers being targeted in clinical trials using fisogatinib to inhibit FGFR4, the receptor for FGF19 (ref. ^25^). By editing the fisogatinib binding site residue 550V to 550M in FGFR4, CRISPR-Select^TIME^ demonstrated that this mutation constitutes a fisogatinib resistance mechanism and that this compound acts through FGFR4 to suppress Hep3B cell proliferation/survival (Fig. 4c).

Furthermore, we applied CRISPR-Select^TIME^ on human MCF7 breast cancer cells to confirm that the estrogen receptor *ESR1*-Y537S mutation, which is emerging as a major resistance mechanism in patients treated with the estrogen antagonist tamoxifen^26,27^, allows estrogen-independent proliferation/survival of MCF7 cells (Fig. 4d).

Finally, we tested CRISPR-Select in primary human organoids, a patient-relevant in vitro system for the modeling of many diseases. We delivered a cassette to human colon epithelial organoids for introduction of KRAS-12D, a recurrent driver of colon cancer^28^. We observed strong selection for KRAS-12D over time, which was further enhanced by the EGF receptor inhibitor gefitinib, reflecting the clinical importance of KRAS-12D mutation as a resistance mechanism for anti-EGF receptor therapy in colon cancer (Fig. 4f). To explore CRISPR-Select modeling of a noncancer disease, we tested loss-of-function variants in *NFKBIZ* in the IL-17A signaling pathway, which have been identified in ulcerative colitis colon epithelium and shown to confer survival advantage to colon epithelial organoids^29,30^. Accordingly, CRISPR-Select demonstrated positive selection for the *NFKBIZ*-E201fs variant in the colon organoids (Fig. 4g).

### In vivo CRISPR-Select^TIME^ variant analysis

**Fig. 5:**
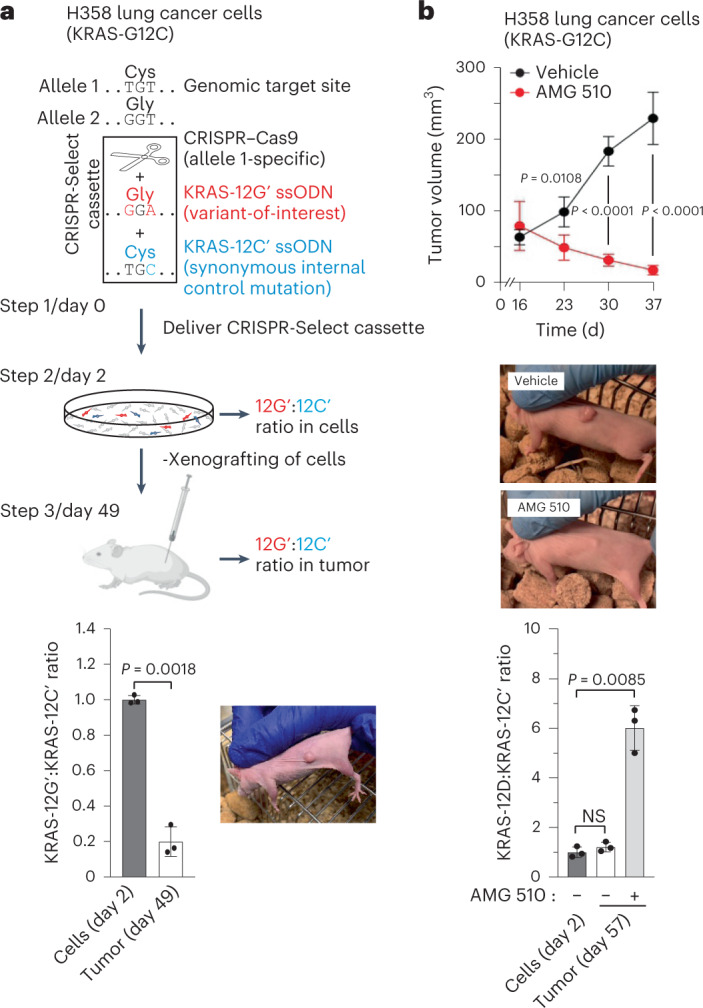
In vivo CRISPR-Select^TIME^ analysis. **a**, A cassette for allele-specific correction of KRAS-12C to 12G′ was delivered to H358 lung cancer cells. On day 2, cells were xenografted into immunocompromised mice and on day 49, selection against KRAS-C12G′ corrected cells in resultant tumors was determined (*n* = 3 cell cultures/mice). *P* value is from a two-tailed paired *t*-test. **b**, H358 cells were CRISPR-Select-edited and xenografted as in (**a**), except that KRAS-12C was mutated to 12D. Two weeks postxenografting, mice were treated with vehicle (−) or AMG 510 (+). Tumor volumes were determined over time and representative mice photographed on day 40. Selection effect of AMG 510 on KRAS-12C′ versus KRAS-12D driver variants in tumors was determined on day 57 (upper panel: *n* = 4 mice per group, *P* values are from two-tailed unpaired *t*-tests; lower panel: *n* = 3 cell cultures/mice per group, *P* values are from two-tailed paired *t*-tests. NS, not significant (*P* > 0.05)). All variant:WT′ or variant:variant′ ratios were normalized to the day 2 value and are means ± s.d. of 3 independent biological replicates.

To determine whether CRISPR-Select allows in vivo functional analysis, we xenografted H358 cells with KRAS-12C corrected to 12G′ into immunocompromised mice (Fig. 5a). The resultant tumors exhibited a ~5-fold depletion of 12G′ cells relative to 12C′ cells. Thus, CRISPR-Select^TIME^ can determine, whether a cancer variant drives tumor formation. We also xenografted H358 cells with KRAS-12C mutated to 12D and treated the mice with AMG 510 or vehicle. In agreement with previous findings^24^, AMG 510 induced overall regression of H358 tumors (Fig. 5b, upper and middle panels). Strikingly, however, within the AMG 510-treated, regressing tumors, cells with KRAS-12D were ~6-fold enriched compared to cells with KRAS-12C′, whereas no enrichment occurred in vehicle-treated tumors (Fig. 5b, lower panel). Thus, also in vivo, CRISPR-Select^TIME^ can define drug resistance mechanisms and determine whether drugs act via their intended target.

### Multiparametric variant analysis by CRISPR-Select^STATE/SPACE^

**Fig. 6:**
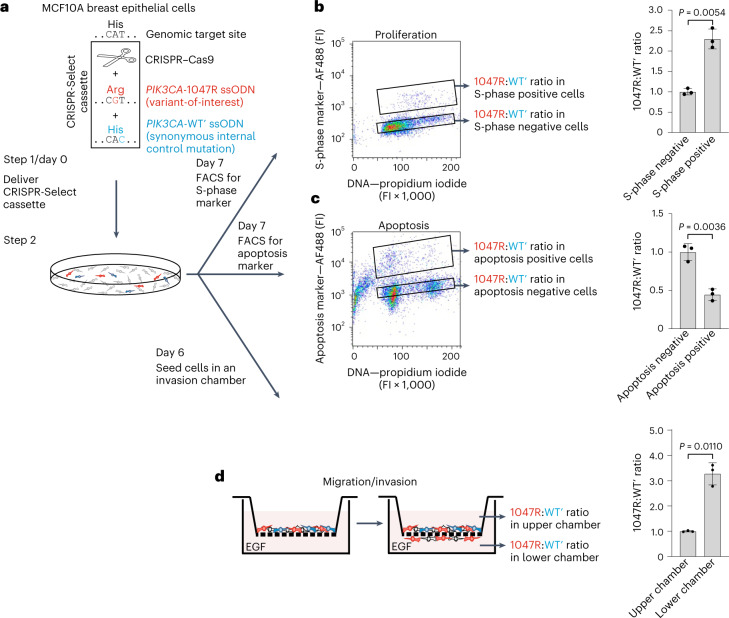
CRISPR-Select^STATE^ and CRISPR-Select^SPACE^ dissection of *PIK3CA-*H1047R effects on cancer hallmarks. **a**, A cassette for *PIK3CA-*H1047R mutation was delivered to iCas9-MCF10A cells. On day 7, cells were subjected to FACS for an S-phase cell state marker (**b**) or an apoptosis cell state marker (**c**). Cell populations positive or negative for the respective cell-state markers were FACS isolated and 1047R:WT′ ratios were determined in the various cell populations. Representative FACS profiles with gating for the sorted S-phase (**b**) or apoptosis (**c**) negative/positive populations are shown. **d**, Alternatively, cells were seeded on day 6 in the upper chamber of a transwell filter insert coated with basement membrane. On day 7, 1047R:WT′ ratios were determined as a function of a spatial dimension, that is, in the cell populations in the upper and the lower chambers. 1047R:WT′ ratios were normalized to the values of (**b**) S-phase negative cells, (**c**) apoptosis negative cells or (**d**) upper chamber cells. Data are means ± s.d. of *n* = 3 independent biological replicates. *P* values are from two-tailed paired *t*-tests. Af488, Alexa Fluor 488; FI, arbitrary fluorescence intensity units.

Tracking variant frequencies as a function of time limits the readout to cell proliferation and/or survival. To vastly expand variant analysis readouts and allow mechanistic dissection of variant effects, we developed CRISPR-Select^STATE^ and CRISPR-Select^SPACE^. We demonstrated the capability of these assay to establish that *PIK3CA-*H1047R confers three cancer hallmarks on MCF10A cells: sustained proliferation, resistance to apoptosis and enhanced migratory and invasive capacities (Fig. 6a-d).

For CRISPR-Select^STATE^, we pulsed iCas9-MCF10A cells transfected with *PIK3CA-*H1047R cassette and cultured in serum/growth factor-depleted medium with 5-ethynyl-2ʼ-deoxyuridine (EdU) to mark cells in the S-phase cell state. Next, we FACS isolated cell populations that were either S-phase positive or negative and determined variant:WT′ ratios in the two populations (Fig. 6b). This revealed enrichment of cells with *PIK3CA-*H1047R in the S-phase positive, relative to the S-phase negative cell population, demonstrating that the variant stimulated proliferation of the cells. In a parallel experiment, CRISPR-Select^STATE^ analysis for the apoptosis marker TUNEL (terminal deoxynucleotidyl transferase dUTP nick end labeling) revealed enrichment of cells with *PIK3CA-*H1047R in the apoptosis negative cell population, demonstrating that the variant conferred resistance to apoptosis (Fig. 6c).

For CRISPR-Select^SPACE^, we seeded iCas9-MCF10A cells transfected with *PIK3CA-*H1047R cassette in the upper chamber of a transwell filter insert (Fig. 6d). The filter of the transwell had been coated with Matrigel basement membrane as an invasion barrier and the lower chamber contained EGF as chemoattractant. The following day, we determined variant:WT′ ratios in the cell population that had remained in the upper chamber and the cell population that had migrated to the lower chamber. This revealed enrichment of cells with *PIK3CA-*H1047R in the lower chamber relative to the upper chamber. CRISPR-Select thereby demonstrated that the variant stimulated the migratory and/or invasive properties of the cells, consistent with the role of PIK3CA as a key mediator of motile/invasive signaling in cells^31^.

**Extended Data Fig. 7:**
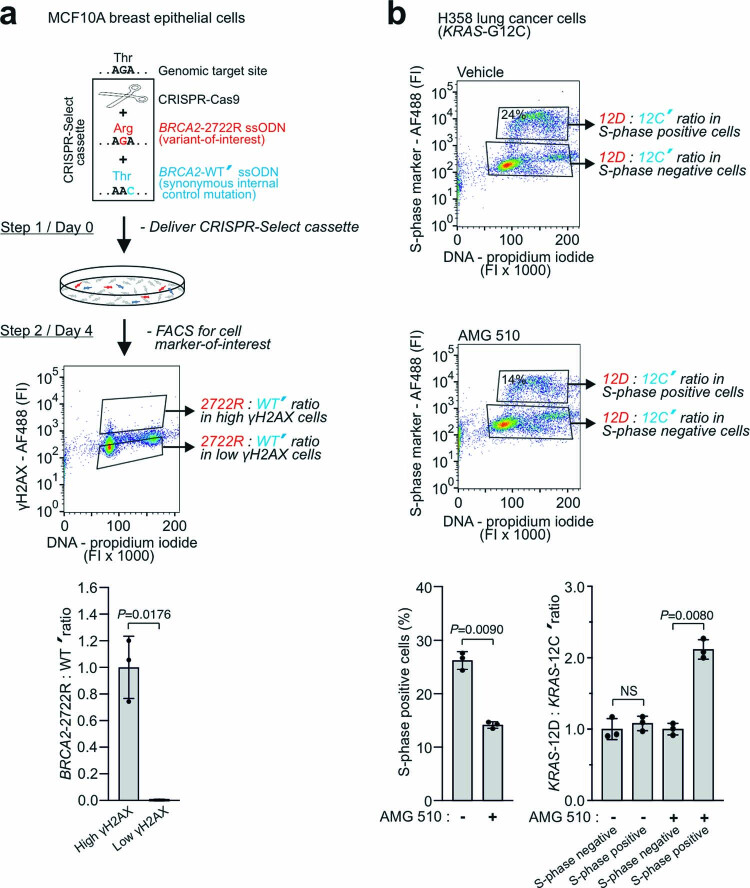
CRISPR-Select-STATE dissection of variant roles in DNA damage and drug resistance mechanisms. **a**, A cassette for *BRCA2-*T2722R mutation was delivered to iCas9-MCF10A-*BRCA2*+/− cells. On day 4, cells were subjected to FACS for isolation of cell populations with high or low levels of the DNA damage marker γH2AX and determination of 2722R:WT′ ratios in the two cell populations. **b**, After delivery of a cassette for KRAS-12C mutation to KRAS-12D in H358 cells, the cells were cultured from day 3 in the presence of vehicle (-) or AMG 510 (+). On day 5, cells were subjected to FACS for an S-phase marker. Lower left panel shows quantification of AMG 510 effect on the percentage of S-phase positive cells, confirming that AMG 510 suppresses proliferation in the bulk cell population. Lower right panel shows 12D:12C´ ratios in the various FACS isolated cell populations, revealing that 12D becomes selectively enriched in S-phase positive cells in the presence of AMG 510. Representative images of the FACS profile with gating for γH2AX low/high (**a**) and S-phase marker (**b**) negative/positive sorted populations are shown. Variant:WT′ (or variant´) ratios were normalized to the values of (**a**) high γH2AX cells or (**b**) S-phase negative cells. Data are means+/− s.d. of *n* = 3 independent biological replicates. P values are from two-tailed paired t-tests. NS, not significant (P > 0.05). AF488; Alexa Fluor 488. FI, arbitrary fluorescence intensity units.

As another example of CRISPR-Select^STATE^, we determined that the *BRCA2-*T2722R pathogenic variant elicits accumulation of the DNA damage marker γH2AX in MCF10A cells (Extended Data Fig. 7a), consistent with the role of BRCA2 in genome maintenance^19^. Finally, we used CRISPR-Select^STATE^ to dissect that the resistance mechanism of KRAS-12D toward AMG 510 in H358 cells involves the ability of KRAS-12D cells to proliferate in the presence of the drug (Extended Data Fig. 7b).

**Extended Data Fig. 8:**
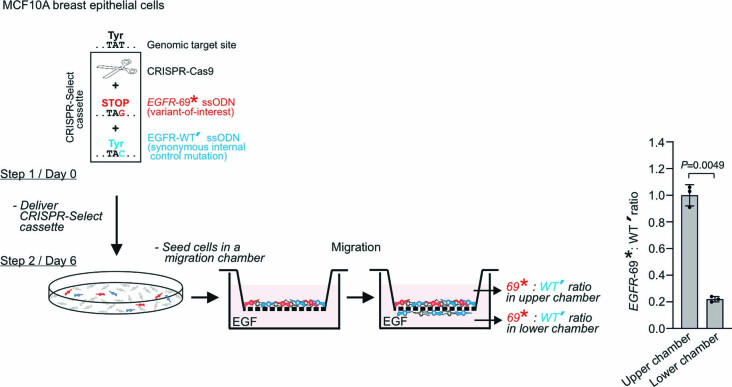
CRISPR-Select^SPACE^ demonstration of EGFR requirement of MCF10A cells for EGF-stimulated chemotactic migration. A cassette for *EGFR-*Y69* mutation was delivered to iCas9-MCF10A cells. On day 6, the cells were seeded in the upper chamber of an uncoated transwell filter insert and on day 7, 69*:WT′ ratios were determined in the cell populations in the upper and lower chambers. The 69*:WTʼ ratios were normalized to the value of the upper chamber cells. The data are means + /− s.d. of *n* = 3 independent biological replicates. P value is from a two-tailed paired t-test.

As a further example of CRISPR-Select^SPACE^, we delivered an *EGFR*-Y69* cassette for inactivation of the EGF receptor to iCas9-MCF10A cells and illustrated the importance of this receptor for EGF-stimulated chemotactic migration in these cells (Extended Data Fig. 8).

### CRISPR-Select^TIME^ 96-well arrayed analysis

**Fig. 7:**
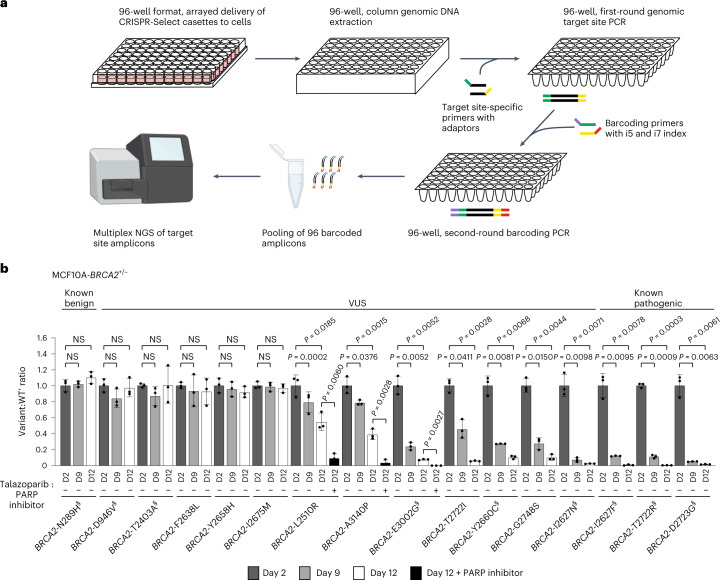
CRISPR-Select^TIME^ 96-well format, arrayed analysis of neutral, small-effect and large-effect *BRCA2* variants at various time points and absence or presence of PARP inhibitor. **a**, Cassettes for various *BRCA2* variants were delivered to iCas9-MCF10A-*BRCA2*^+/−^ cells in 96-well plate, which were cultured for the time periods and in the presence of vehicle or PARP inhibitor. Thereafter, genomic DNA extraction and first and second-round PCR were performed in 96-well plate formats to obtain barcoded amplicons of the genomic target sites. Finally, amplicons were pooled and subjected to NGS. **b**, Variant:WT′ ratios were determined and normalized to the day 2 value. Double S (§) indicates variants that were analyzed in the 96-well assay as well as in single-dish assays in this study. Data are means ± s.d. of *n* = 3 replicate transfections. *P* values are from two-tailed paired *t*-tests. NS, not significant (*P* > 0.05).

Finally, we adopted CRISPR-Select^TIME^ to 96-well plate format for higher throughput arrayed variant analysis. Specifically, we transfected iCas9-MCF10A-*BRCA2*^+/−^ cells with various *BRCA2* variant cassettes in a 96-well plate and cultured the cells until two end time points, followed by 96-well genomic DNA extraction, target site PCR, amplicon library preparation and finally, amplicon NGS (Fig. 7a). The 96-well results were fully concordant with those obtained in single-dish assays for the variants tested in both formats (indicated with double S (§) in Fig. 7b). The only difference was that selection against loss-of-function variants occurred slightly faster in 96-well format, likely because cultures were split more frequently, which reduces epithelial cell island formation, favoring proliferation. The 96-well-arrayed format allowed parallel analysis of many variants under several culture conditions (for example, culture periods and drugs), revealing variant effects that ranged from none to large. Of note, small-effect *BRCA2* variants manifested more profoundly in the presence of PARP inhibitor or with longer culture time, which may provide one means to better reveal their effect.

## Discussion

We developed CRISPR-Select as an accurate assay for cell phenotypes elicited by genetic sequence variants and for dissection of the underlying mechanisms. The method is highly versatile, allowing variant analysis in any desired cell type and facile modification for diverse applications, such as FACS-based cell state readouts, in vivo studies and 96-well higher throughput screens.

CRISPR-Select^TIME^ shares key advantages with previous CRISPR-based knock-in^12^ and base editing^13,14^ screening approaches for determining variant effects on proliferation and/or survival as follows: (1) variant analysis in proper genomic context, thereby avoiding artifacts associated with approaches of overexpressed variant cDNA, and (2) variant analysis in a cell population, which provides fast results, allows the study of loss-of-function variants in essential genes and minimizes artifacts from clonal variation.

CRISPR-Select^SPACE^ and CRISPR-Select^STATE^ greatly expand functional variant analysis beyond the cell proliferation/survival readout of previous CRISPR approaches for cell population-based variant analysis. CRISPR-Select^STATE^ can determine effects of variants on any physiological state or biochemical process of a cell with a FACS marker, thereby allowing dissection of mechanism(s) of variant effects. Furthermore, we estimate that CRISPR-Select^STATE^ will enable cell population-based variant analysis for a majority of the genes underlying the 5,000–8,000 human monogenic diseases^15^, few of which impact cell proliferation/survival and therefore could not be studied by the previous cell population-based CRISPR approaches.

The present arrayed assay differs fundamentally from the previous multiplexed variant screening assays regarding the target site PCR/NGS analysis. The PCR primers for the knock-in multiplexed screening assays annealed to engineered sites in the repair template to obtain sufficient sequence coverage for knock-in alleles^12^. Thereby, however, only knock-in, but not WT alleles are quantified, and absolute frequencies of variants in the cell population cannot be determined. The PCR primers for the base editing multiplexed screening assay annealed to the virally inserted gRNA construct^13,14^. Thereby, the target site is not analyzed and loss/gain-of-variant frequencies (that is, functional effects) are measured indirectly, as it is not known whether the desired editing has occurred.

By contrast, the annealing of CRISPR-Select PCR primers outside the edited region provides complete characterization of all alleles in the sample, which allows validation that the desired editing occurred as well as calculation of absolute frequencies of cells with variant and WT′ to validate that sufficient numbers of cells underlie the analysis. When such validated analysis is performed at two points in time, space or state, the effect of the variant on the analyzed cell parameter can be conclusively determined. Furthermore, the complete allele characterization also allows assessment of whether ‘built-in loss of heterozygosity’ has occurred, indicated by a high proportion of frameshift InDels relative to knock-in events in the cell population. We demonstrated at the single-cell level that ‘built-in loss of heterozygosity’ is an inherent feature of CRISPR-Select with the implication that the method works robustly for loss-of-function variants in recessive genes in diploid cells. CRISPR-Select single-cell analysis experiments may also be performed with much higher cell throughput using the Tapestri system but at high sequencing costs^32^. Finally, the complete allele characterization allows assessment of selection for/against the internal frameshift InDel control to validate that apparently neutral variants are truly neutral. The knock-in screening assays^12^ can also control for this latter point, as any loss-of-function variants in the screening assay can serve same function as our frameshift InDel control.

We typically obtain day 2 knock-in frequencies from 2% to 10% without prior cassette screening, and the method works well with day 2 knock-in down to 1–2%. This tracks the fate of 170–340 independent knock-in cell clones, which are covered by a sufficient number of reads with the typical 30,000–50,000 reads per target site. The Supplementary Note provides guidelines to estimate how many reads are needed with a given knock-in frequency and effect size. Even with day 2 knock-in frequencies of 0.6–0.7%, variant effects may be determined accurately (Extended Data Fig. 6).

**Extended Data Fig. 9:**
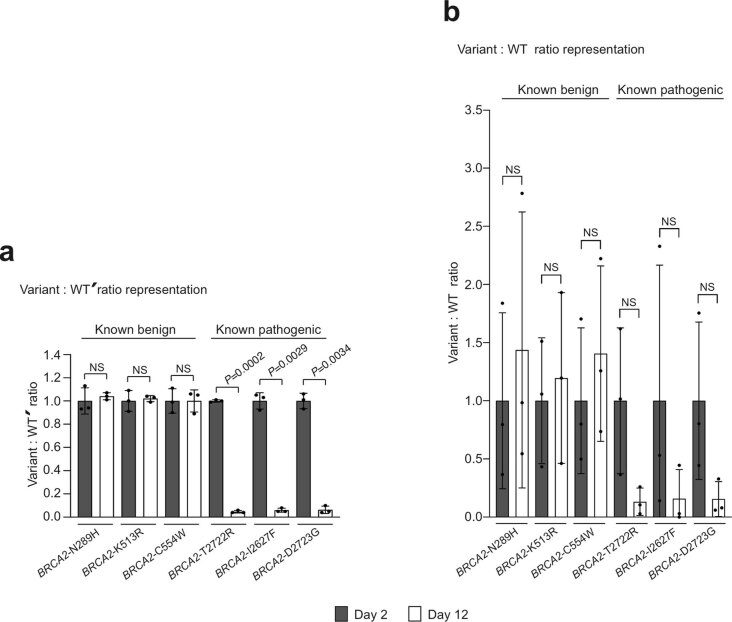
The internal WT′ control normalizes out experimental variation to yield more conclusive results. CRISPR-Select^TIME^ analysis of known benign or pathogenic *BRCA2* missense variants with variant frequencies shown as ratios to **a**, WT′ alleles or to **b**, WT alleles. Data are from Fig. 3a). All variant:WT′ or variant:WT ratios were normalized to the day 2 value. Data are means+/− s.d. of *n* = 3 independent biological replicates. P values are from two-tailed paired t-tests. NS, not significant (P > 0.05).

In summary, high accuracy and reliability are key characteristics of CRISPR-Select due to the following several features: (1) the cassette and arrayed assay design creates a well-controlled assay with internal normalization standard (WT′) for determining variant effects. WT′ effectively normalizes out experimental confounders, such as potential CRISPR off-target effects, varying transfection efficiency/toxicity, cell density, edge effects, etc., because cells with variant and WT′ will be affected the same way by the confounders and therefore, any difference in phenotype will be due to the effect of the variant. Extended Data Fig. 9 provides an example of the advantage of normalizing variant frequencies to WT′, as opposed to WT alleles, (2) determination of absolute variant and WT′ frequencies (accurately quantified by amplicon NGS) in the cell population at the two assay points and an analysis based on differences in variant:WT′ ratios, (3) ‘built-in loss of heterozygosity’ feature to reveal loss-of-function variants and complete characterization of editing outcomes to confirm that it has occurred, (4) the frameshift InDel control that apparently neutral variants are truly neutral, (v) with the typical knock-in frequencies obtained, data are based on hundreds-to-thousands of knock-in cells, effectively diluting out artifacts caused by cell heterogeneity in the targeted cell population, and (5) variant analysis occurs in proper genomic/cellular context.

Currently, CRISPR-Select has some limitations. The method is not suitable for multi-loci editing, partly because the NGS analysis cannot determine whether the variants were introduced in the same cell. Furthermore, while multiplex CRISPR-Select analysis of several adjacent variants in a gene may be possible using same gRNA and WT′, the number will be limited to three to four variants, because knock-in frequency per variant will decrease with increasing number of variants included. Finally, CRISPR-Select is less suited for analysis of VUS in promoters and similar noncoding regions, where neutrality of WT′ typically cannot be predicted.

In conclusion, CRISPR-Select can determine virtually any functional effect of a coding or splice site variant in different cell types with accuracy and reliability. The combined set of quality and quantitation controls may surpass alternative functional variant assays and a CRISPR-Select result generally does not need validation by other assays. Single-well CRISPR-Select analysis of a potential disease variant found in a patient is therefore highly suited for ad hoc molecular diagnosis in the clinic. Furthermore, 96-well arrayed CRISPR-Select allows characterization of multiple disease-linked variants; notably, with the same high data quality as the single-well assays. Altogether, CRISPR-Select may therefore provide a useful functional variant assay for research, diagnostics and drug development in genetic diseases.

## Methods

The use of human colon organoids for research was approved by the Scientific Ethics Committee of the Copenhagen Capital Region. The patient provided informed written consent to the protocol (H-18005342). Animal experiments complied with the regulations and were approved by the Danish Experimental Inspectorate (2019-15-0201-00307).

### Cells and culture conditions

MCF10A cells (ATCC, CRL-10317) were cultured in Dulbecco’s modified Eagle medium/F12, HEPES (Thermo Fisher Scientific, 31330038) supplemented with 5% (vol/vol) horse serum (Thermo Fisher Scientific, 26050088), 10 μg ml^−1^ insulin (Sigma, I1882), 20 ng ml^−1^ EGF (Peprotech, AF-100-15), 0.5 μg ml^−1^ hydrocortisone (Sigma, H0888) and 100 ng ml^-1^ cholera toxin (Sigma, C8052). H358 (=NCI-H358) cells (ATCC, CRL-5807) were cultured in Roswell Park Memorial Institute 1640 medium (ATCC, 30-2001) supplemented with 10% (vol/vol) fetal bovine serum (Thermo Fisher Scientific, 12389802). Hep3B cells (ATCC, HB-8064) were cultured in Minimum Essential Medium (Thermo Fisher Scientific, 41090028) supplemented with 10% (vol/vol) fetal bovine serum, 1% (vol/vol) minimal essential medium nonessential amino acids (Thermo Fisher Scientific, 11140035) and 1 mM sodium pyruvate (Thermo Fisher Scientific, 11360070). MCF7 cells (ATCC, HTB-22) were cultured in Dulbecco’s modified Eagle medium (Thermo Fisher Scientific, 31966021) supplemented with 10% (vol/vol) fetal bovine serum. HEK 293T (ATCC, CRL-3216) cells were cultured in Dulbeccoʼs modified Eagle medium, high glucose supplemented with 10% (vol/vol) fetal bovine serum.

Human colon epithelial organoids were generated from colon tissue from a 54 years old healthy woman at Herlev Hospital, Denmark, as described^33^. The organoids were maintained as described^34^ with minor modifications. Organoids were cultured in a 50:50 mix of Cultrex UltiMatrix Reduced Growth Factor Basement Membrane Extract (R&D Systems, BME001-01) and Advanced Dulbecco’s modified Eagle medium/Hamʼs F12 (Thermo Fisher Scientific, 12634010) supplemented with 10 mM HEPES (Thermo Fisher Scientific, 15630056), 2 mM GlutaMAX (Thermo Fisher Scientific, 35050061), B-27 supplement (Thermo Fisher Scientific, 12587010), 10 nM gastrin (Sigma, G9145), 1 mM N-acetylcysteine (Sigma, A9165), 500 nM A83-01 (Tocris, 2939), 100 ng ml^−1^ human IGF-1 (BioLegend, 590906), 50 ng ml^−1^ human FGF2 (Peprotech, 100-18B), 100 ng ml^−1^ human Noggin (PeproTech, 120-10C), 50 ng ml^−1^ human EGF, 1 μg ml^−1^ human R-spondin-1 (R&D Systems, 4645-RS) and 100 ng ml^−1^ mouse Wnt3a (R&D Systems, 1324-WN-002). The culture medium was refreshed every 2 days. Organoids were passaged once a week by sequential mechanical disruption with a P1000 and a P200 pipette tip.

All media were supplemented with 1% (vol/vol) penicillin/streptomycin (Thermo Fisher Scientific, 15070063).

### iCas9-MCF10A*-BRCA2*^+/+^ and *-BRCA2*^+/−^ cells

An iCas9-MCF10A-*BRCA2*^+/+^ clonal cell line with Cas9 expressed from stably integrated TRE3G Edit-R Inducible Lentiviral Cas9 construct (Horizon, CAS11229) was a gift from Roderick L. Beijersbergen, The Netherlands Cancer Institute. To generate *BRCA2*^+/−^ cells, iCas9-MCF10A^+/+^ cells were transfected with dual gRNAs targeting intron 1 of *BRCA2* (*BRCA2* intron 1) and intergenic sequence 3′ to the *BRCA2* gene (*BRCA2* 3′ intergenic) (see Extended Data Fig. 4 and Supplementary Table 1 and below for MCF10A editing). Three days post-transfection, cells were plated singly into 96-well plates using a FACS Aria III instrument (BD Biosciences) and expanded to clonal cell lines. The clones were genotyped by genomic PCR with primer pairs specific for wild-type or *BRCA2* deletion alleles (Supplementary Table 3) and agarose electrophoresis of PCR products that were also analyzed by Sanger sequencing.

An iCas9-MCF10A cell pool was generated by first sub-cloning iCas9 into the lentiviral vector pCW57-GFP-2A that allows doxycycline-induced co-expression of green fluorescent protein (GFP) and a coding sequence inserted in the multiple cloning site after the 2A sequence (Addgene, 71783). Briefly, Q5 High-Fidelity 2× Master Mix (New England Biolabs, M0492S) was used to PCR amplify the coding sequence of Cas9 from the pSpCas9-2A-GFP (Addgene, 48138) and the entire pCW57-GFP-2A vector using primers to introduce overlapping overhangs in both PCR products. Next, the two PCR products were recombined using the NEBuilder HiFi DNA Assembly Master Mix (New England Biolabs, E2621S) to produce pCW57-iGFP-2A-Cas9 that was confirmed by sanger sequencing and deposited with Addgene (plasmid 170805). For production of lentivirus containing iGFP-2A-Cas9, 2.5 × 10^6^ HEK 293T cells were plated in a 58-cm^2^ dish and the following day cotransfected with 7 μg pCW57-iGFP-2A-Cas9 transfer plasmid, 5 μg VSVG envelope plasmid and 6 μg PAX8 packaging plasmid using Lipofectamine 3000 (Thermo Fisher Scientific, L3000001) according to manufacturerʼs protocol. Twenty-four hours post-transfection, the virus was concentrated from supernatant through ultracentrifugation, resuspended in MCF10A cell culture medium and added to MCF10A cells plated the previous day at 2.0 × 10^6^ cells in a 58-cm^2^ dish. After 7 h, medium was changed and at 36 h, 1 μg ml^−1^ doxycycline (Sigma, D3447) was added to the medium to induce GFP-2A-Cas9 expression. After 5 days, a pool of stably transduced cells was FACS isolated based on GFP expression. Two more rounds of culture for 20 days and FACS isolation for GFP-positive cells were performed to produce the final iCas9-MCF10A cell pool. All MCF10A experiments used the clonal iCas9-MCF10A cell line, except the CRISPR-Select^SPACE^ experiment that used the iCas9-MCF10A cell pool.

### CRISPR-Select cassette design

CRISPR-Select cassettes were designed by first selecting a gRNA for *Streptococcus pyogenes* Cas9 with the online software Benchling (https://benchling.com), for which the base pairs to be mutated were located as close as possible to the genomic cut site to promote efficient knock-in and within the PAM or the one to ten PAM proximal nucleotides within the gRNA target site (the seed region) for the mutations to destroy the Cas9 target site^35^. This relatively broad window allows identification of a Cas9-gRNA for the majority of variants. If a Cas9-gRNA cannot be found, another CRISPR tool may be used from the expanding repertoire of available CRISPR systems^36^. As a further gRNA criterion, the closest potential off-target site must have at least 1 bp mismatch in the PAM or seed region. SsODN repair templates were designed such that the synonymous WT′ mutation was placed at the same position as, or within one to three nucleotides from, the variant of interest to promote knock-in at similar frequencies (for location of WT′ for splice site variants, see Extended Data Fig. 5b). For WT′, the Human Splicing Finder online tool was used to assess that the mutation did not create a splice site (http://www.umd.be/HSF3/)^37^ and the Codon Usage Database was consulted to check that the mutation did not generate a rarely used codon in the edited species (http://www.kazusa.or.jp/codon)^38^. Polarity of ssODNs and gRNAs was chosen based on the following rules delineated by Paix et al. (ref. ^39^): if mutations in ssODN repair templates were located >4 base pairs from the cut site, sense ssODNs were used for mutations located to the left of the DNA double-strand break and antisense ssODNs for mutations located to the right of the break, otherwise ssODN polarity was not considered. Polarity of gRNAs was not considered in any case. The length of ssODN homology arms was 45 nucleotides, based on ref. ^40^. Lists of all gRNAs and ssODNs used are given in Supplementary Tables 1 and 2.

### CRISPR-Select cassette delivery

gRNAs were used in the form of crRNA:tracrRNA duplexes purchased from Integrated DNA Technologies and reconstituted in nuclease-free duplex buffer at 10 or 100 μM. For ribonucleoprotein (RNP) generation, Alt-R SpCas9 Nuclease V3 from Integrated DNA Technologies (1081059) was used. ssODNs were purchased as unmodified Ultramer DNA oligonucleotides at 100 μM in IDTE, pH 8.0 from Integrated DNA Technologies.

For iCas9-MCF10A cells, Cas9 expression was induced by adding 1 μg ml^−1^ doxycycline to the culture medium 24 h before transfection of 50–70% confluent cells with the remainder of the cassette. Briefly, for a 9.6-cm^2^ well, 75 pmol each of crRNA and tracrRNA in 7.5 µl were mixed and allowed to complex by incubation for 10 min at room temperature. Next, 125 µl OptiMEM (Thermo Fisher Scientific, 31985062) were added, and then 10 pmol each of the variant and WT′ ssODN in 2 µl were added and the solution was mixed. Finally, the nucleotide solution was mixed with 7.5 µl Lipofectamine RNAiMAX (Thermo Fisher Scientific, 13778) in 125 µl OptiMEM, incubated for 10 min at room temperature and dripped onto iCas9-MCF10A cells in fresh medium and doxycycline. For other culture area sizes, the amounts of reagents were adjusted proportionally.

For H358, Hep3B and MCF7 cells, the cassette was delivered as RNP and ssODNs by nucleofection in a Lonza 4D-Nucleofector device, using the following Lonza Cell Line 4D-Nucleofector kit/pulse program: H358, SF/CM-130; Hep3B, SF/EH-100; MCF7, SE/EN-130. Briefly, for a nucleofection of 10^6^ cells, 250 pmol each of crRNA and tracrRNA were mixed and allowed to complex by incubation for 10 min at room temperature. Next, 62 pmol Cas9 proteins were mixed with the crRNA:tracrRNA duplexes and incubated for further 10 min. Next, cells were resuspended in 20 μl of electroporation solution and added to RNPs and 120 pmol each of variant and WT′ ssODN. Finally, the cell suspension was transferred to a nucleocuvette and electroporated using the relevant pulse program.

For human colon epithelial organoids, the cassette was delivered as RNP and ssODNs by electroporation using a NEPA21 electroporation device (Nepa Gene), as described^34^ with minor modifications. In brief, 24 h before electroporation, the culture medium of proliferative organoids was supplemented with 10 μM Y-27632 (Selleck Chemicals, S1049), 5 μM CHIR99021 (Sigma, 361559) and 1.25% (vol/vol) DMSO. On the day of electroporation, organoids were dissociated into clumps containing 5–15 cells by sequential mechanical dissociation in PBS + 0.1% BSA with a P1000 and a P200 pipette tip, followed by dissociation with TrypLE (Thermo Fisher Scientific, 12605010) supplemented with 10 μM Y-27632 for 8 min at 37 °C. For the electroporation of 3 × 10^5^ cells, 500 pmol each of crRNA and tracrRNA were mixed and allowed to complex by incubation for 10 min at room temperature. Next, 153 pmol Cas9 protein were mixed with the crRNA:tracrRNA duplexes and incubated for further 10 min. Next, cells were resuspended in 70 μl OptiMEM and added to RNPs and 600 pmol each of variant and WT′ ssODN. Finally, the cell suspension was transferred to a cuvette and electroporated using the pulse program described in ref. ^34^. After electroporation, 500 µl Advanced Dulbeccoʼs modified Eagle medium/Hamʼs F12 was added to the cuvette and the cells were left for 30 min at room temperature. Then, cells were plated at 1 × 10^5^ cells per well in a 48-well plate with complete culture medium supplemented with 10 μM Y-27632 and 5 μM CHIR99021 for the first 2 days after electroporation.

### In vitro CRISPR-Select^TIME^

#### All assays

On day 2 (or four for organoids), after delivery of CRISPR-Select cassette, an aliquot of the relevant cell population was collected for the early time point variant:WT′ analysis. Another portion of the cell population was replated according to the gene and cell type analyzed, as follows:

#### BRCA2 assays

For cell culture dish assays, iCas9-MCF10A cells were seeded at 50,000–70,000 into 58-cm^2^ dishes with complete culture medium. On day 7, cells were trypsinated and 50,000–100,000 of the cells replated into a new dish and cultured until collecting on day 12. For, 96-well plate assays, the cells were seeded at ~10,000 per well and cultured for 2–3 days until confluency, where after the cells were trypsinated and split 1:3, which was continued until collecting the cells at a confluent state. When indicated, cells were treated with 0.1% (vol/vol) DMSO vehicle or 2 nM talazoparib (Axon Medchem, 2502).

#### PIK3CA and PTEN assays

iCas9-MCF10A cells were seeded at ~30% confluency into a 58-cm^2^ dish. After 16 h, cells were washed with phosphate-buffered saline and the medium was changed to culture medium with omission of serum and any supplements for PIK3CA assays or to same medium, but without phenol red (Thermo Fisher Scientific, 11039021) and supplemented with insulin for *PTEN* assays.

#### KRAS assays in H358 cells

H358 cells were seeded at 20% confluency into 58-cm^2^ dishes with complete culture medium and, depending on the experiment, 0.1% (vol/vol) DMSO vehicle or 0.12 μM AMG 510 (MedChemExpress, HY-114277).

#### FGFR4 assays

Hep3B cells were seeded at 20% confluency into a 6-well plate and cultured in complete medium in the presence of 0.1% (vol/vol) DMSO vehicle or 0.72 μM fisogatinib (=BLU-554) (MedChemExpress, HY-100492). The medium was changed every day due to low inhibitor stability.

#### *ESR1* assays

MCF7 cells were seeded at 20% confluency into a 9.6-cm^2^ dish with culture medium without phenol red (Thermo Fisher Scientific, 21063029) and with charcoal/dextran treated serum (Cytiva, SH30068.01).

#### KRAS and *NFKBIZ* assays in colon organoids

Organoids were dissociated into single cells by incubation with TrypLE supplemented with 10 μM Y-27632 for 20 min at 37 °C. For KRAS, the organoids were next cultured in the absence of EGF and the absence or presence of 1 μM gefitinib (Selleck Chemicals, S5098). For *NFKBIZ*, the organoids were cultured in the absence of Noggin. The organoids were not passaged during the assays and the culture medium was refreshed every 2 days.

For all assays, unless otherwise indicated, culture medium was changed every three days and cells were split to ~20% confluency, when 70–80% confluency was reached. After cell splitting in PIK3CA/PTEN experiments under starvation culture conditions, cells were replated in complete medium to allow cell attachment and the following day washed with phosphate-buffered saline and then incubated in starvation culture medium. At indicated time points, cells were collected for variant:WT′ analysis.

### In vivo CRISPR-Select^TIME^

Mice were housed in an environmentally controlled room (temperature 23 ± 2 °C, relative humidity 50 ± 20%) on a 12-h light/12-h dark cycle. On day 2 after delivery of CRISPR-Select cassette to H358 cells, an aliquot of the cell population was collected for variant:WT′ analysis. Another portion of the cell population was injected subcutaneously as a cell suspension of 3 × 10^5^ cells in 0.1 mL of a 1:1 (vol/vol) mix of H358 cell culture medium and matrigel (Corning, 356234) into the left flank of 4–5-week-old weight-matched athymic female mice (Charles River Laboratories, strain code 490) (*n* = 8 mice). For the KRAS-G12D experiment, mice were randomly distributed into two groups on day 16, which received a daily oral dose by gavage of either vehicle or AMG 510 (100 mg kg^−1^) formulated in 2% (vol/vol) hydroxypropyl methylcellulose and 1% (vol/vol) Tween 80 (n = 4 mice per group) until end of the experiment. Tumor volume was monitored once a week using a caliper and calculated by the following modified ellipsoidal formula: length × width^2^ × 0.52. The maximum tumor size limit of 12 mm in diameter was followed. At indicated end points, tumors were collected. DNA was extracted from an aliquot of whole tumor minced in phosphate-buffered saline using a TissueLyser LT instrument (Qiagen, 85600) and subjected to variant:WT′ analysis.

### CRISPR-Select^SPACE^

After delivery of CRISPR-Select cassette, iCas9-MCF10A cells were cultured for 6 days in complete culture medium. Thereafter, 1.1 × 10^6^ cells were seeded in culture medium with 0.1% (wt/vol) bovine serum albumin (Sigma, A8412) but omission of serum and other supplements in the upper chamber of 4.7 cm^2^, and 8 µm pore size polycarbonate filter transwell chambers (Corning, 3428), either precoated with 12 μg ml^−1^ growth-factor-reduced basement membrane extract (R&D Systems, 3533-001-02) for thin-layer extracellular matrix invasion assay^41^ for PIK3CA or left uncoated for EGFR assays. Next, cells were allowed to migrate/invade against 30 nM EGF in culture medium without serum and other supplements in the lower chamber for 16 h for PIK3CA assays or for 32 h for *EGFR* assays. Thereafter, cells on the upper surface of the filter were collected with a cell scraper, while cells on the lower surface were collected by submersion of the transwell in trypsin solution for 30 min followed by cell scraping. Finally, both cell populations were collected for variant:WT′ analysis.

### CRISPR-Select^STATE^

After delivery of CRISPR-Select cassette, cells were treated according to the given gene, cell type and cell state analyzed, described below.

#### PIK3CA proliferation assay

On day 2, 1.5 × 10^6^ iCas9-MCF10A cells were seeded in a 145-cm^2^ dish. On day 3, cells were washed with phosphate-buffered saline and incubated in culture medium without serum or any supplements. On day 7, S-phase cells were pulse-labeled by incubation for 2 h with 10 μM of the thymidine analog 5-ethynyl-2 deoxyuridine (EdU), where after all cells in the dish were collected and prepared for flow cytometrical detection and FACS isolation of S-phase cells, using the Click-iT EdU assay (Thermo Fisher Scientific, C10425) according to the manufacturerʼs instructions. Briefly, cells were fixed and permeabilized, and the incorporated EdU was labeled with Alexa Fluor 488 azide by click chemistry. Furthermore, DNA was stained with propidium iodide solution containing RNase.

#### KRAS proliferation assay

From day 3, sister cultures were cultured in the presence of 0.1% (vol/vol) DMSO vehicle or 0.12 μM AMG 510. On day 5, cells were labeled with EdU and prepared for flow cytometrical detection of S-phase cells, as described above for PIK3CA.

#### PIK3CA apoptosis assay

On day 2, 2.5 × 10^6^ iCas9-MCF10 cells were seeded in a 145-cm^2^ dish. On day 3, cells were washed with phosphate-buffered saline and incubated in culture medium without serum or any supplements. On day 7, all cells in the dish were collected and prepared for flow cytometrical detection and FACS isolation of apoptotic cells, using the APO-BrdU terminal deoxynucleotidyl transferase dUTP nick end labeling (TUNEL) assay (Thermo Fisher Scientific, A23210) according to the manufacturerʼs instructions. Briefly, cells were fixed/permeabilized by incubation with ice-cold 70% ethanol for 16 h. Next, DNA fragments were labeled with deoxythymidine analog 5-bromo-2′-deoxyuridine 5′-triphosphate (BrdUTP) and stained with Alexa Fluor 488-labeled anti-BrdU antibody. Total DNA was stained using propidium iodide solution containing RNase.

#### BRCA2 DNA damage assay

After 4 days of growth in normal culture medium, iCas9-MCF10 cells were prepared for flow cytometrical detection and FACS isolation of cells with the DNA damage marker γH2AX^42^. Briefly, 2.0 × 10^6^ cells were fixed in 70% ethanol and incubated in 0.25% (vol/vol) triton X-100 in PBS for 10 min at room temperature. After washing, cells were incubated with a mouse monoclonal pSer139-H2AX antibody (Millipore, 05-636) for 1 h at room temperature, followed by washing and incubation with an Alexa Fluor 488-labeled secondary antibody (Invitrogen, A11001) for 30 min at room temperature.

For all assays, samples were subjected to flow cytometry in a BD FACSMelody instrument (BD Bioscience) using FACSChorus software. Cell populations were gated for relevant levels of the cell state marker of interest and isolated by FACS. Data were analyzed using FlowJo software (version 10.4).

### CRISPR-Select variant:WT′ analysis

Genomic DNA was extracted from CRISPR-Select-edited cell populations using the following: (1) GenElute Mammalian Genomic DNA Miniprep Kit (Sigma, G1N350-1KT) for samples with >100,000 cells, (2) Quick-DNA Microprep Plus Kit (Zymo Research, D4074) for samples with <100,000 cells and (3) Quick-DNA 96 Plus Kit (Zymo Research, D4071) for 96-well plate samples. For all PCRs, 100 ng genomic DNA was used as a template, except for the apoptosis and γ-H2AX assays that used 50 ng. Primer pairs for PCR amplification of the target site (Supplementary Table 3) were designed to anneal 40–120 nt outside the region covered by the ssODN repair donors and to generate PCR products of 230–350 bps, using Primer-BLAST^43^ from NCBI (https://www.ncbi.nlm.nih.gov/tools/primer-blast/). To prepare the PCR products for amplicon NGS, a previously reported, two-round PCR procedure was used^44^. For the first-round PCR, the target-site-specific primers contained overhangs with binding sites for the second-round primer pairs. The PCR was performed in a total volume of 25 µl, containing 0.3 μM of each primer and 12.5 µl of Phusion U Green Multiplex PCR Master Mix (Thermo Fisher Scientific, F564L) and PCR conditions were as follows: initial denaturing for 1 min at 98 °C, then 35 cycles of 98 °C for 10 s, 60 °C for 30 s (reducing the temperature by 0.1 °C each cycle), 72 °C for 15 s and a final post-PCR extension for 5 min at 72 °C. In the second-round PCR, primers contained overhangs with sample-specific barcodes as well as adaptors for NGS. As template, 2.5 μl of the first-round PCR was used in a total PCR volume of 12.5 µl, containing 0.3 μM of each primer, and 6.25 µl of Phusion U Green Multiplex PCR Master Mix (Thermo Fisher Scientific, F564L). Second-round PCR conditions were as follows: initial denaturing for 30 s at 98 °C, then 8 cycles of 98 °C for 10 s, 60 °C for 30 s, 72 °C for 15 s and a final post-PCR extension for 2 min at 72 °C. After mixing roughly equal amounts of PCR products, the amplicon sequencing library was made by using the MiSeq Reagent Kit v2 (Illumina, MS-102-2002) and finally sequenced in a MiSeq instrument from Illumina, according to manufacturerʼs instructions. Sequencing depths ranged from 20,000 to 200,000 reads per sample. NGS data were analyzed by the CRISPResso2 online tool using default settings (https://crispresso.pinellolab.partners.org/submission)^45^ and have been deposited with the NCBI Sequence Read Archive database with the accession number PRJNA759404.

### Single-cell target site Sanger sequencing

On day 2 after delivery of CRISPR-Select cassette, single iCas9-MCF10A cells were sorted using a FACSMelody (BD Bioscience) instrument into each well of 96-well PCR plates containing 3 µl of QuickExtract DNA extraction solution (Lucigen, QE 09050) per well. Plates were vortexed and centrifuged after sorting. Next, genomic DNA was extracted by incubating the plates for 25 min at 65 °C and for 5 min at 95 °C. Then, 20 µl PCR mixture composed of Phusion U Green Multiplex PCR Master Mix (Thermo Fisher Scientific, F564S) and 0.2 µM PCR primers (*BRCA2-*T2722R-SingleCell-F/R; Supplementary Table 3) were added to each well. PCR was performed using the following cycle conditions: initial denaturation for 1 min at 98 °C for 1 min, then 35 cycles of 98 °C for 10 s; 65 °C for 30 s, 72 °C for 15 s and a final post-PCR extension for 5 min at 72 °C. PCR products were checked by 1.5% (wt/vol) agarose gel electrophoreses and then Sanger sequenced by GENEWIZ using the sequencing primer *BRCA2-*T2722R-SingleCell-Seq (Supplementary Table 3). Sequencing results were analyzed using the ICE-Analysis online tool (https://ice.synthego.com)^46^.

### Statistical analysis

We used two-tailed paired *t*-tests to calculate the significance in all cases, except Fig. 5b tumor graphs, where we employed a two-tailed unpaired *t*-test. *P* ≤ 0.05 was considered significant. Data distribution was assumed to be normal, but this was not tested. We used GraphPad Prism 9 (GraphPad Prism version 9.2.0 for Windows, GraphPad Software, www.graphpad.com) to generate the data plots.

### Reporting summary

Further information on research design is available in the Nature Portfolio Reporting Summary linked to this article.

## Online content

Any methods, additional references, Nature Portfolio reporting summaries, source data, extended data, supplementary information, acknowledgements, peer review information; details of author contributions and competing interests; and statements of data and code availability are available at 10.1038/s41588-022-01224-7.

## Supplementary information

Supplementary Information Supplemental Tables 1–3 and Supplemental Note.

Reporting Summary

## Data Availability

All data sets are available within the article and/or deposited with the NCBI Sequence Read Archive database with the accession number PRJNA759404. Data on BRCA2 variants from ClinVar were accessed through https://www.ncbi.nlm.nih.gov/clinvar/. For CRISPR-Select cassette design, the Human Splicing Finder online tool was accessed through http://www.umd.be/HSF3/ and the Codon Usage Database was accessed through http://www.kazusa.or.jp/codon. Primer-BLAST was used to design primer pairs for PCR amplification of the target sites and was accessed through https://www.ncbi.nlm.nih.gov/tools/primer-blast/.

## Code availability

All computational tools used are published. No custom code or mathematical algorithms were used in this study.
