# Supplementary material for: Multiparametric and accurate functional analysis of genetic sequence variants using CRISPR-Select
Source: Nat Genet. 2022 Dec 5;54(12):1983–93. doi: 10.1038/s41588-022-01224-7 (PMC9729100; doi:10.1038/s41588-022-01224-7)
Supplement: Supplementary file 1 — Supplemental Tables 1–3 and Supplemental Note. [file 41588_2022_1224_MOESM1_ESM.pdf]

nature genetics

Technical Report https://doi.org/10.1038/s41588-022-01224-7

Multiparametric and accurate functional analysis of genetic sequence variants using CRISPR-Select

In the format provided by the
authors and unedited

Supplemental Table 1. Sequence of crRNAs.

crRNA name	20 nt guide sequence of crRNA
PIK3CA-H1047R	CAAATGAATGATGCACATCA
PTEN-L182*	AATCCAGATGATTCTTTAAC
BRCA2 intron1	AGGCGGCGTTGGTCTCTAAC
BRCA2 3′ intergenic	AGACAATAGCAGTTGTACCA
BRCA2-N289H	ACTTCATCTTCTAGGACATT
BRCA2-K513R	CTTGCATTGAAAGTCTCTTT
BRCA2-C554W	ATCAATTAAATTTGGACATA
BRCA2-D946V	AGTGTCAATTAAAAAAGATT
BRCA2-T2403A	GGTGGAACAAAGACTTTGGT
BRCA2-L2510R	TTTGCAAGATACAGACTGCC
BRCA2-I2627F	ATCACTATAGATGGATCATA
BRCA2-I2627N	ATCACTATAGATGGATCATA
BRCA2-F2638L	TATGGAATGTGCCTTTCCTA
BRCA2-Y2658H	TGCTTCTTCAACTAAAATAC
BRCA2-Y2660C	TTTCACTTTTAGATATGATA
BRCA2-I2675M	ATCGGCTATAAAAAAGATAA
BRCA2-T2722I	CCATTATTGAACTTACAGAT
BRCA2-T2722R	CCATTATTGAACTTACAGAT
BRCA2-D2723G	TTATTGAACTTACAGATGGG
BRCA2-G2748S	AAGAATGGCAGACTGACAGT
BRCA2-E3002G	TTATATTCTCTGTTAACAGA
BRCA2-A3140P	GGCCTTCTTACTTTATTTGC
BRCA2-67+1G>C	AAGACACGCTGCAACAAAGC
BRCA2-316+1G>T	GATAAATTCAAATTAGACTT
BRCA2-476-2A>G	TCCCCTTTTTTTACCCCCAG
BRCA2-517-2A>G	TTTTGGTGTCTGACGACCCT
KRAS-C12D/G	GTAGTTGGAGCTTGTGGCGT
FGFR4-V550M	GCACTCCACGATCACGTACA
ESR1-Y537S	TCCAGCAGCAGGTCATAGAG
EGFR-Y69*	ATCATAATTCCTCTGCACAT
NFKBIZ-E201fs	ACCAACGCCCGGGGAGAGCA

All crRNAs are *H. sapiens* specific.

Supplemental Table 2. Sequence of ssODN repair templates.

ssODN name	ssODN sequence	
Reference sequence	AGCAAGAGGCTTTGGAGTATTTCATGAAACAAATGAATGATGCACATCATGGTGGCTGGACAACAAAAATGGATTGGATCTTCCACACAAT	His
PIK3CA-H1047H (WT´)	AGCAAGAGGCTTTGGAGTATTTCATGAAACAAATGAATGATGCACACCATGGTGGCTGGACAACAAAAATGGATTGGATCTTCCACACAAT	His
PIK3CA-H1047R (Variant)	AGCAAGAGGCTTTGGAGTATTTCATGAAACAAATGAATGATGCACGTCATGGTGGCTGGACAACAAAAATGGATTGGATCTTCCACACAAT	Arg
Reference sequence	TATTCCCAGTCAGAGGCGCTATGTGTATTATTATAGCTACCTGTTAAAGAATCATCTGGATTATAGACCAGTGGCACTGTTGTTTCACAA	Leu
PTEN-L182L (WT´)	TATTCCCAGTCAGAGGCGCTATGTGTATTATTATAGCTACCTGTTGAAGAATCATCTGGATTATAGACCAGTGGCACTGTTGTTTCACAA	Leu
PTEN-L182* (Variant)	TATTCCCAGTCAGAGGCGCTATGTGTATTATTATAGCTACCTGTAAAAGAATCATCTGGATTATAGACCAGTGGCACTGTTGTTTCACAA	STOP
Reference sequence	TTTAAAGTAAATAGCTGCAAAGACCACATTGGAAAGTCAATGCCAAATGTCCTAGAAGATGAAGTATATGAAACAGTTGTAGATACCTCTGAA	Asn
BRCA2-N289N (WT´)	TTTAAAGTAAATAGCTGCAAAGACCACATTGGAAAGTCAATGCCAAACGTCCTAGAAGATGAAGTATATGAAACAGTTGTAGATACCTCTGAA	Asn
BRCA2-N289H (Variant)	TTTAAAGTAAATAGCTGCAAAGACCACATTGGAAAGTCAATGCCACATGTCCTAGAAGATGAAGTATATGAAACAGTTGTAGATACCTCTGAA	His
Reference sequence	TTCAGGGTATCAAAAAGTCTATATTCAGAATAAGAGAATCACCTAAAGAGACTTTCAATGCAAGTTTTTCAGGTCATATGACTGATCCAAAC	Lys
BRCA2-K513K (WT´)	TTCAGGGTATCAAAAAGTCTATATTCAGAATAAGAGAATCACCTAAGGAGACTTTCAATGCAAGTTTTTCAGGTCATATGACTGATCCAAAC	Lys
BRCA2-K513R (Variant)	TTCAGGGTATCAAAAAGTCTATATTCAGAATAAGAGAATCACCTAGAGAGACTTTCAATGCAAGTTTTTCAGGTCATATGACTGATCCAAAC	Arg
Reference sequence	ACTGGAAATACATACTGTTTGCTCACAGAAGGAGGACTCCTTATGTCCAAATTTAATTGATAATGGAAGCTGGCCAGCCACCACCACACAG	Cys
BRCA2-C554C (WT´)	ACTGGAAATACATACTGTTTGCTCACAGAAGGAGGACTCCTTATGCCCAAATTTAATTGATAATGGAAGCTGGCCAGCCACCACCACACAG	Cys
BRCA2-C554W (Variant)	ACTGGAAATACATACTGTTTGCTCACAGAAGGAGGACTCCTTATGGCCAAATTTAATTGATAATGGAAGCTGGCCAGCCACCACCACACAG	Trp
Reference sequence	GGAGACACAGGTGATAAACAAGCAACCCAAGTGTCAATTAAAAAAGATTTGGTTTATGTTCTTGCAGAGGAGAACAAAAATAGTGTAAAGCAG	Asp
BRCA2-D946D (WT´)	GGAGACACAGGTGATAAACAAGCAACCCAAGTGTCAATTAAAAAAGACTTGGTTTATGTTCTTGCAGAGGAGAACAAAAATAGTGTAAAGCAG	Asp
BRCA2-D946V (Variant)	GGAGACACAGGTGATAAACAAGCAACCCAAGTGTCAATTAAAAAAGTTTTGGTTTATGTTCTTGCAGAGGAGAACAAAAATAGTGTAAAGCAG	Val
Reference sequence	ACAAGAAATGAAAAAATGAGACACTTGATTACTACAGGCAGACCAACCAAAGTCTTTGTTCCACCTTTTAAAACTAAATCACATTTTCACAGA	Thr
BRCA2-T2403T (WT´)	ACAAGAAATGAAAAAATGAGACACTTGATTACTACAGGCAGACCAACAAAAGTCTTTGTTCCACCTTTTAAAACTAAATCACATTTTCACAGA	Thr
BRCA2-T2403A (Variant)	ACAAGAAATGAAAAAATGAGACACTTGATTACTACAGGCAGACCAGCCAAAGTCTTTGTTCCACCTTTTAAAACTAAATCACATTTTCACAGA	Ala
Reference sequence	ATTAAGAAGAAACAAAGGCAACGCGTCTTTCCACAGCCAGGCAGTCTGTATCTTGCAAAAACATCCACTCTGCCTCGAATCTCTCTGAAAGCA	Leu
BRCA2-L2510L (WT´)	ATTAAGAAGAAACAAAGGCAACGCGTCTTTCCACAGCCAGGCAGTTTGTATCTTGCAAAAACATCCACTCTGCCTCGAATCTCTCTGAAAGCA	Leu
BRCA2-L2510R (Variant)	ATTAAGAAGAAACAAAGGCAACGCGTCTTTCCACAGCCAGGCAGTCGGTATCTTGCAAAAACATCCACTCTGCCTCGAATCTCTCTGAAAGCA	Arg
Reference sequence	CCAAAGCTTATTTCTAGAATTTGGGTTTATAATCACTATAGATGGATCATATGGAAACTGGCAGCTATGGAATGTGCCTTTCCTAAGGAATTT	Ile
BRCA2-I2627I (WT´)	CCAAAGCTTATTTCTAGAATTTGGGTTTATAATCACTATAGATGGATTATATGGAAACTGGCAGCTATGGAATGTGCCTTTCCTAAGGAATTT	Ile
BRCA2-I2627F (Variant)	CCAAAGCTTATTTCTAGAATTTGGGTTTATAATCACTATAGATGGTTCATATGGAAACTGGCAGCTATGGAATGTGCCTTTCCTAAGGAATTT	Phe
Reference sequence	CCAAAGCTTATTTCTAGAATTTGGGTTTATAATCACTATAGATGGATCATATGGAAACTGGCAGCTATGGAATGTGCCTTTCCTAAGGAATTT	Ile
BRCA2-I2627I (WT´)	CCAAAGCTTATTTCTAGAATTTGGGTTTATAATCACTATAGATGGATTATATGGAAACTGGCAGCTATGGAATGTGCCTTTCCTAAGGAATTT	Ile
BRCA2-I2627N (Variant)	CCAAAGCTTATTTCTAGAATTTGGGTTTATAATCACTATAGATGGAACATATGGAAACTGGCAGCTATGGAATGTGCCTTTCCTAAGGAATTT	Asn
Reference sequence	CACTATAGATGGATCATATGGAAACTGGCAGCTATGGAATGTGCCTTTCCTAAGGAATTTGCTAATAGATGCCTAAGCCCAGAAAGGGTGCTT	Phe
BRCA2-F2638F (WT´)	CACTATAGATGGATCATATGGAAACTGGCAGCTATGGAATGTGCCTTCCCTAAGGAATTTGCTAATAGATGCCTAAGCCCAGAAAGGGTGCTT	Phe
BRCA2-F2638L (Variant)	CACTATAGATGGATCATATGGAAACTGGCAGCTATGGAATGTGCCTTGCCTAAGGAATTTGCTAATAGATGCCTAAGCCCAGAAAGGGTGCTT	Leu
Reference sequence	GCTAATAGATGCCTAAGCCCAGAAAGGGTGCTTCTTCAACTAAAATACAGGCAAGTTTAAAGCATTACATTACGTAATCATATACGGCAGTAT	Tyr
BRCA2-Y2658Y (WT´)	GCTAATAGATGCCTAAGCCCAGAAAGGGTGCTTCTTCAACTAAAATATAGGCAAGTTTAAAGCATTACATTACGTAATCATATACGGCAGTAT	Tyr
BRCA2-Y2658H (Variant)	GCTAATAGATGCCTAAGCCCAGAAAGGGTGCTTCTTCAACTAAAACACAGGCAAGTTTAAAGCATTACATTACGTAATCATATACGGCAGTAT	His
Reference sequence	AGAGTCACACTTCCTAAAATATGCATTTTTGTTTTCACTTTTAGATATGATACGGAAATTGATAGAAGCAGAAGATCGGCTATAAAAAAGATA	Tyr
BRCA2-Y2660Y (WT´)	AGAGTCACACTTCCTAAAATATGCATTTTTGTTTTCACTTTTAGATACGATACGGAAATTGATAGAAGCAGAAGATCGGCTATAAAAAAGATA	Tyr
BRCA2-Y2660C (Variant)	AGAGTCACACTTCCTAAAATATGCATTTTTGTTTTCACTTTTAGATGTGATACGGAAATTGATAGAAGCAGAAGATCGGCTATAAAAAAGATA	Cys

Supplemental Table 2. Sequence of ssODN repair templates (continued).

ssODN name		ssODN sequence
Reference sequence	Ile	TATGATACGGAAATTGATAGAAGCAGAAGATCGGCTATAAAAAAGATAATGGAAAGGGATGACACAGCTGCAAAAACACTTGTTCTCTGTGTT
BRCA2-I2675I (WT´)	Ile	TATGATACGGAAATTGATAGAAGCAGAAGATCGGCTATAAAAAAGATCATGGAAAGGGATGACACAGCTGCAAAAACACTTGTTCTCTGTGTT
BRCA2-I2675M (Variant)	Met	TATGATACGGAAATTGATAGAAGCAGAAGATCGGCTATAAAAAAGATGATGGAAAGGGATGACACAGCTGCAAAAACACTTGTTCTCTGTGTT
Reference sequence	Thr	AAACTAGTAGTGCAGATACCCAAAAAGTGGCCATTATTGAACTTACAGATGGGTGGTATGCTGTTAAGGCCCAGTTAGATCCTCCCCTCTTA
BRCA2-T2722T (WT´)	Thr	AAACTAGTAGTGCAGATACCCAAAAAGTGGCCATTATTGAACTTACGGATGGGTGGTATGCTGTTAAGGCCCAGTTAGATCCTCCCCTCTTA
BRCA2-T2722I (Variant)	Ile	AAACTAGTAGTGCAGATACCCAAAAAGTGGCCATTATTGAACTTATAGATGGGTGGTATGCTGTTAAGGCCCAGTTAGATCCTCCCCTCTTA
Reference sequence	Thr	AAACTAGTAGTGCAGATACCCAAAAAGTGGCCATTATTGAACTTACAGATGGGTGGTATGCTGTTAAGGCCCAGTTAGATCCTCCCCTCTTA
BRCA2-T2722T (WT´)	Thr	AAACTAGTAGTGCAGATACCCAAAAAGTGGCCATTATTGAACTTACGGATGGGTGGTATGCTGTTAAGGCCCAGTTAGATCCTCCCCTCTTA
BRCA2-T2722R (Variant)	Arg	AAACTAGTAGTGCAGATACCCAAAAAGTGGCCATTATTGAACTTAGAGATGGGTGGTATGCTGTTAAGGCCCAGTTAGATCCTCCCCTCTTA
Reference sequence	Asp	CTAGTAGTGCAGATACCCAAAAAGTGGCCATTATTGAACTTACAGATGGGTGGTATGCTGTTAAGGCCCAGTTAGATCCTCCCCTCTTAGCT
BRCA2-D2723D (WT´)	Asp	CTAGTAGTGCAGATACCCAAAAAGTGGCCATTATTGAACTTACAGACGGGTGGTATGCTGTTAAGGCCCAGTTAGATCCTCCCCTCTTAGCT
BRCA2-D2723G (Variant)	Gly	CTAGTAGTGCAGATACCCAAAAAGTGGCCATTATTGAACTTACAGGTGGGTGGTATGCTGTTAAGGCCCAGTTAGATCCTCCCCTCTTAGCT
Reference sequence	Thr Gly	GATCCTCCCCTCTTAGCTGTCTTAAAGAATGGCAGACTGACAGTTGGTCAGAAGATTATTCTTCATGGAGCAGAACTGGTGGGCTCTCCTGAT
BRCA2-G2748G (WT´)	Thr	GATCCTCCCCTCTTAGCTGTCTTAAAGAATGGCAGACTGACCGTTGGTCAGAAGATTATTCTTCATGGAGCAGAACTGGTGGGCTCTCCTGAT
BRCA2-G2748S (Variant)	Ser	GATCCTCCCCTCTTAGCTGTCTTAAAGAATGGCAGACTGACAGTTAGTCAGAAGATTATTCTTCATGGAGCAGAACTGGTGGGCTCTCCTGAT
Reference sequence	Thr Glu	CTGAGTATTTGGCGTCCATCATCAGATTTATATTCTCTGTTAACAGAAGGAAAGAGATACAGAATTTATCATCTTGCAACTTCAAAATCTAAA
BRCA2-T3001T (WT´)	Thr	CTGAGTATTTGGCGTCCATCATCAGATTTATATTCTCTGTTAACCGAAGGAAAGAGATACAGAATTTATCATCTTGCAACTTCAAAATCTAAA
BRCA2-E3002G (Variant)	Gly	CTGAGTATTTGGCGTCCATCATCAGATTTATATTCTCTGTTAACAGGAGGAAAGAGATACAGAATTTATCATCTTGCAACTTCAAAATCTAAA
Reference sequence	Phe Ala	CTCCAGTGGCGACCAGAATCCAAATCAGGCCTTCTTACTTTATTTGCTGGAGATTTTTCTGTGTTTTCTGCTAGTCCAAAAGAGGGCCACTTT
BRCA2-A3140A (WT´)	Phe	CTCCAGTGGCGACCAGAATCCAAATCAGGCCTTCTTACTTTATTCGCTGGAGATTTTTCTGTGTTTTCTGCTAGTCCAAAAGAGGGCCACTTT
BRCA2-A3140P (Variant)	Pro	CTCCAGTGGCGACCAGAATCCAAATCAGGCCTTCTTACTTTATTTCCTGGAGATTTTTCTGTGTTTTCTGCTAGTCCAAAAGAGGGCCACTTT
Reference sequence	Lys	GGCCAACATTTTTTGAAATTTTTAAGACACGCTGCAACAAAGCAGGTATTGACAAATTTTATATAACTTTATAAATTACACCGAGA
BRCA2-K21K (WT´)	Lys	GGCCAACATTTTTTGAAATTTTTAAGACACGCTGCAACAAGGCAGGTATTGACAAATTTTATATAACTTTATAAATTACACCGAGA
BRCA2-67+1G>C (Variant)		GGCCAACATTTTTTGAAATTTTTAAGACACGCTGCAACAAAGCAGCTATTGACAAATTTTATATAACTTTATAAATTACACCGAGA
Reference sequence	Asp	ACCAATCTCCTGTAAAAGAATTAGATAAATTCAAATTAGACTTAGGTAAGTAATGCAATATGGTAGACTGGGGAGAACTACAAACT
BRCA2-N104N (WT´)	Asp	ACCAATCTCCTGTAAAAGAATTAGATAAATTCAAATTAGATTTAGGTAAGTAATGCAATATGGTAGACTGGGGAGAACTACAAACT
BRCA2-316+1G>T (Variant)		ACCAATCTCCTGTAAAAGAATTAGATAAATTCAAATTAGACTTAGTTAAGTAATGCAATATGGTAGACTGGGGAGAACTACAAACT
Reference sequence	Val	GTTAATAAAAATAAAACTTAACAATTTTCCCCTTTTTTTACCCCCAGTGGTATGTGGGAGTTTGTTTCATACACCAAAGTTTGTGAAGGTAAAT
BRCA2-V159V (WT´)	Val	GTTAATAAAAATAAAACTTAACAATTTTCCCCTTTTTTTACCCCCAGTCGTATGTGGGAGTTTGTTTCATACACCAAAGTTTGTGAAGGTAAAT
BRCA2-476-2A>G (Variant)		GTTAATAAAAATAAAACTTAACAATTTTCCCCTTTTTTTACCCCCGGTGGTATGTGGGAGTTTGTTTCATACACCAAAGTTTGTGAAGGTAAAT
Reference sequence	Arg	TGATCAGGGCATTTCTATAAAAAATAAACTATTTTCTTTCCTCCCAGGGTCGTCAGACACCAAAACATATTTCTGAAAGTCTAGGAGCTGA
BRCA2-R174R (WT´)	Arg	TGATCAGGGCATTTCTATAAAAAATAAACTATTTTCTTTCCTCCCAGGGTCGCCAGACACCAAAACATATTTCTGAAAGTCTAGGAGCTGA
BRCA2-517-2A>G (Variant)		TGATCAGGGCATTTCTATAAAAAATAAACTATTTTCTTTCCTCCCGGGGTCGTCAGACACCAAAACATATTTCTGAAAGTCTAGGAGCTGA
Reference sequence	Cys	GGCCTGCTGAAAATGACTGAATATAAACTTGTGGTAGTTGGAGCTTGTGGCGTAGGCAAGAGTGCCTTGACGATACAGCTAATTCAGAATCAT
KRAS-C12C´ (Variant´)	Cys	GGCCTGCTGAAAATGACTGAATATAAACTTGTGGTAGTTGGAGCTTGCGGCGTAGGCAAGAGTGCCTTGACGATACAGCTAATTCAGAATCAT
KRAS-C12G´ (WT´)	Gly	GGCCTGCTGAAAATGACTGAATATAAACTTGTGGTAGTTGGAGCTGGAGGCGTAGGCAAGAGTGCCTTGACGATACAGCTAATTCAGAATCAT
KRAS-C12D (Variant)	Asp	GGCCTGCTGAAAATGACTGAATATAAACTTGTGGTAGTTGGAGCTGATGGCGTAGGCAAGAGTGCCTTGACGATACAGCTAATTCAGAATCAT

Supplemental Table 2. Sequence of ssODN repair templates (continued).

ssODN name	Annotated codons	ssODN sequence
Reference sequence	Val	GGGCCCGCAGGAACTCCCGCAGGTTTCCCTTGGCGGCGCACTCCACGATCACGTACAGGGGCCCTGCAGAGGGAGTGGAGGGAGCGTGGA
FGFR4-V550V (WT´)	Val	GGGCCCGCAGGAACTCCCGCAGGTTTCCCTTGGCGGCGCACTCAACGATCACGTACAGGGGCCCTGCAGAGGGAGTGGAGGGAGCGTGGA
FGFR4-V550M (Variant)	Met	GGGCCCGCAGGAACTCCCGCAGGTTTCCCTTGGCGGCGCACTCCATGATCACGTACAGGGGCCCTGCAGAGGGAGTGGAGGGAGCGTGGA
Reference sequence	Tyr	GGAGCATCTGTACAGCATGAAGTGCAAGAACGTGGTGCCCCTCTATGACCTGCTGCTGGAGATGCTGGACGCCCACCGCCTACATGCGCC
ESR1-Y537Y (WT´)	Tyr	GGAGCATCTGTACAGCATGAAGTGCAAGAACGTGGTGCCCCTCTACGACCTGCTGCTGGAGATGCTGGACGCCCACCGCCTACATGCGCC
ESR1-Y537S (Variant)	Ser	GGAGCATCTGTACAGCATGAAGTGCAAGAACGTGGTGCCCCTCTCTGACCTGCTGCTGGAGATGCTGGACGCCCACCGCCTACATGCGCC
Reference sequence	Tyr	GTTCAATAACTGTGAGGTGGTCCTTGGGAATTTGGAAATTACCTATGTGCAGAGGAATTATGATCTTTCCTTCTTAAAGGTTGGTGACTT
EGFR-Y69Y (WT´)	Tyr	GTTCAATAACTGTGAGGTGGTCCTTGGGAATTTGGAAATTACCTACGTGCAGAGGAATTATGATCTTTCCTTCTTAAAGGTTGGTGACTT
EGFR-Y69* (Variant)	STOP	GTTCAATAACTGTGAGGTGGTCCTTGGGAATTTGGAAATTACCTAGGTGCAGAGGAATTATGATCTTTCCTTCTTAAAGGTTGGTGACTT
Reference sequence	Ser; Glu Asp Val His Leu Asp Glu Pro	TCTTTCCAGACACCACCTCAAACACCAACGCCCGGGGAGAGCATGGAAGATGTTCATCTCAATGAACCCAAACAGGAGAGCAGTGCTGAT
NFKBIZ-E201E (WT´)	Ser; Glu Asp Val His Leu Asp Glu Pro	TCTTTCCAGACACCACCTCAAACACCAACGCCCGGGGAGAGTATGGAGGATGTTCATCTCAATGAACCCAAACAGGAGAGCAGTGCTGAT
NFKBIZ-E201fs (Variant)	Ser; Glu Glu Arg Cys Ser Ser Glu STOP	TCTTTCCAGACACCACCTCAAACACCAACGCCCGGGGAGAGTATGGAAGAAAGATGTTCATCTCAATGAACCCAAACAGGAGAGCAGTGCTGAT

The codon affected by mutagenesis is boxed. WT´ nucleotide and amino acid substitutions are indicated in blue fond. Variant nucleotide and amino acid substitutions are indicated in red fond. The crRNA target site is underlined in the reference sequence. The PAM is underlined and indicated in purple fond in the reference sequence. The position of the CRISPR-Cas9 elicited DNA double-strand break is indicated by a vertical

Supplemental Table 3. Sequence of PCR primers.

Primers used in first round PCR for amplicon sequencing

Primer name	Primer sequence
PIK3CA-H1047R-F	*ACACTCTTTCCCTACACGACGCTCTTCCGATCT*AGAGGCTTTGGAGTATTTCATGA
PIK3CA-H1047R-R	*TGACTGGAGTTCAGACGTGTGCTCTTCCGATCT*GTCTTTGCCTGCTGAGAGTT
PTEN- L182*-F	*ACACTCTTTCCCTACACGACGCTCTTCCGATCT*ACGACCCAGTTACCATAGCA
PTEN- L182*-R	*TGACTGGAGTTCAGACGTGTGCTCTTCCGATCT*AGTGCCACTGGTCTATAATCCA
BRCA2-N289H-F	*ACACTCTTTCCCTACACGACGCTCTTCCGATCT*TACTTTAACAGGATTTGGAAAAACA
BRCA2-N289H-R	*TGACTGGAGTTCAGACGTGTGCTCTTCCGATCT*AGATTTTTCACATTCATCAGCGT
BRCA2-K513R-F	*ACACTCTTTCCCTACACGACGCTCTTCCGATCT*AAGAGAGATGAAGAGCAGCAT
BRCA2-K513R-R	*TGACTGGAGTTCAGACGTGTGCTCTTCCGATCT*GAGTCCTCCTTCTGTGAGCAA
BRCA2-C554W-F	*ACACTCTTTCCCTACACGACGCTCTTCCGATCT*TCAGAATAAGAGAATCACCTAAAGA
BRCA2-C554W-R	*TGACTGGAGTTCAGACGTGTGCTCTTCCGATCT*AGTGGATATTAAACCTGCATTCT
BRCA2-D946V-F	*ACACTCTTTCCCTACACGACGCTCTTCCGATCT*AACAAGCAACCCAAGTGTCA
BRCA2-D946V-R	*TGACTGGAGTTCAGACGTGTGCTCTTCCGATCT*AGAGTCCTGCCCATTTGTTC
BRCA2-T2403A-F	*ACACTCTTTCCCTACACGACGCTCTTCCGATCT*TGACTTTGGAAAAATCTTCAAGC
BRCA2-T2403A-R	*TGACTGGAGTTCAGACGTGTGCTCTTCCGATCTCT*GTTCAACTCTGTGAAAATGTGA
BRCA2-L2510R-F	*ACACTCTTTCCCTACACGACGCTCTTCCGATCT*ACAAGTCTTCAGAATGCCAGAGA
BRCA2-L2510R-R	*TGACTGGAGTTCAGACGTGTGCTCTTCCGATCT*ACACTCTGTCATAAAAGCCATCA
BRCA2-I2627F-F	*ACACTCTTTCCCTACACGACGCTCTTCCGATCT*TGAATTCAGTATCATCCTATGTGGT
BRCA2-I2627F-R	T*GACTGGAGTTCAGACGTGTGCTCTTCCGATCT*CAGAAACCTTAACCATACTGCCG
BRCA2-I2627N-F	*ACACTCTTTCCCTACACGACGCTCTTCCGATCT*GGTGTGGATCCAAAGCTTATTTC
BRCA2-I2627N-R	*TGACTGGAGTTCAGACGTGTGCTCTTCCGATCT*TGTAATGCTTTAAACTTGCCTGT
BRCA2-F2638L-F	*ACACTCTTTCCCTACACGACGCTCTTCCGATCT*GGTGTGGATCCAAAGCTTATTTC
BRCA2-F2638L-R	*TGACTGGAGTTCAGACGTGTGCTCTTCCGATCT*TGTAATGCTTTAAACTTGCCTGT
BRCA2-Y2658H-F	*ACACTCTTTCCCTACACGACGCTCTTCCGATCT*ACTGGCAGCTATGGAATGTGC
BRCA2-Y2658H-R	*TGACTGGAGTTCAGACGTGTGCTCTTCCGATCT*AGACTACACAGAAACCTTAACCA
BRCA2-Y2660C-F	*ACACTCTTTCCCTACACGACGCTCTTCCGATCT*GGAATTCTAGAGTCACACTTCCT
BRCA2-Y2660C-R	*TGACTGGAGTTCAGACGTGTGCTCTTCCGATCT*TTTTGCAGCTGTGTCATCCC
BRCA2-I2675M-F	*ACACTCTTTCCCTACACGACGCTCTTCCGATCT*GGAATTCTAGAGTCACACTTCCT
BRCA2-I2675M-R	*TGACTGGAGTTCAGACGTGTGCTCTTCCGATCT*TTTTGCAGCTGTGTCATCCC
BRCA2-T2722I-F	*ACACTCTTTCCCTACACGACGCTCTTCCGATCT*CTGACATAATTTCATTGAGCGCA
BRCA2-T2722I-R	*TGACTGGAGTTCAGACGTGTGCTCTTCCGATCT*CCACCAGTTCTGCTCCATGA
BRCA2-T2722R-F	*ACACTCTTTCCCTACACGACGCTCTTCCGATCT*CTGACATAATTTCATTGAGCGCA
BRCA2-T2722R-R	*TGACTGGAGTTCAGACGTGTGCTCTTCCGATCT*CCACCAGTTCTGCTCCATGA
BRCA2-D2723G-F	*ACACTCTTTCCCTACACGACGCTCTTCCGATCT*GATGACACAGCTGCAAAAACAC
BRCA2-D2723G-R	*TGACTGGAGTTCAGACGTGTGCTCTTCCGATCT*GCTTCAAGAGGTGTACAGGCA
BRCA2-G2748S-F	*ACACTCTTTCCCTACACGACGCTCTTCCGATCT*TGGTATGCTGTTAAGGCCCA

BRCA2-G2748S-R	*TGACTGGAGTTCAGACGTGTGCTCTTCCGATCT*TGACTGATTTTTACCAAGAGTGC
BRCA2-E3002G-F	*ACACTCTTTCCCTACACGACGCTCTTCCGATCT*TGCATCTTTCTCATCTTTCTCCA
BRCA2-E3002G-R	*TGACTGGAGTTCAGACGTGTGCTCTTCCGATCT*TTGTCGCTGCTAACTGTATGT
BRCA2-A3140P-F	*ACACTCTTTCCCTACACGACGCTCTTCCGATCT*GGACTTGCCCCTTTCGTCTAT
BRCA2-A3140P-R	*TGACTGGAGTTCAGACGTGTGCTCTTCCGATCT*TGTCTCTTGAAAGTGGCCCTC
BRCA2-67+1G>C-F	*ACACTCTTTCCCTACACGACGCTCTTCCGATCT*TGGATCCAAAGAGAGGCCAAC
BRCA2-67+1G>C-R	*TGACTGGAGTTCAGACGTGTGCTCTTCCGATCT*TGTGGTTAACCTGCAAACGA
BRCA2-316+1G>T-F	*ACACTCTTTCCCTACACGACGCTCTTCCGATCT*TCTGCCGCTGTACCAATCTC
BRCA2-316+1G>T-R	*TGACTGGAGTTCAGACGTGTGCTCTTCCGATCT*AGAGACTGATTTGCCCAGCA
BRCA2-476-2A>G-F	*ACACTCTTTCCCTACACGACGCTCTTCCGATCT*ACCTAAGGGATTTGCTTTGTTTTA
BRCA2-476-2A>G-R	*TGACTGGAGTTCAGACGTGTGCTCTTCCGATCT*TTGGTGTATGAAACAAACTCCCAC
BRCA2-517-2A>G-F	*ACACTCTTTCCCTACACGACGCTCTTCCGATCT*TCCTTAATGATCAGGGCATTTCT
BRCA2-517-2A>G-R	*TGACTGGAGTTCAGACGTGTGCTCTTCCGATCT*ACCTCATCTGCTCTTTCTTGT
KRAS-C12-F	*ACACTCTTTCCCTACACGACGCTCTTCCGATCT*GGTACTGGTGGAGTATTTGATAGTG
KRAS-C12-R	*TGACTGGAGTTCAGACGTGTGCTCTTCCGATCT*ACCTCTATTGTTGGATCATATTCGT
FGFR4-V550M-F	*ACACTCTTTCCCTACACGACGCTCTTCCGATCT*CTCTCCACGCTCCCTCCA
FGFR4-V550M-R	*TGACTGGAGTTCAGACGTGTGCTCTTCCGATCT*ACTCCAGATACTGCATGCCT
ESR1-Y537S-F	*ACACTCTTTCCCTACACGACGCTCTTCCGATCT*TTCCCCTTCTAGGGATTTCAGC
ESR1-Y537S-R	*TGACTGGAGTTCAGACGTGTGCTCTTCCGATCT*GTGGGCGTCCAGCATCTC
EGFR-Y69*-F	*ACACTCTTTCCCTACACGACGCTCTTCCGATCT*TCAATAACTGTGAGGTGGTCC
EGFR-Y69*-R	*TGACTGGAGTTCAGACGTGTGCTCTTCCGATCT*TTCAAGTGGAATTCTGCCCA
NFKBIZ-E201fs-F	*ACACTCTTTCCCTACACGACGCTCTTCCGATCT*TTTCCTTCTTTCCAGACACCAC
NFKBIZ-E201fs-R	*TGACTGGAGTTCAGACGTGTGCTCTTCCGATCT*GTTCAGGGAAACGGGGCTG

Other primers

**Primer name**	**Primer sequence**
BRCA2-T2722R-SingleCell-F	TGATACGGAAATTGATAGAAGCAGA
BRCA2-T2722R-SingleCell-R	GGGCTTCAAGAGGTGTACAGG
BRCA2-T2722R-SingleCell-seq	ATTGATAGAAGCAGAAGATCGGC
BRCA2-1F	GTGTTTTACAGCTGCTGGGC
BRCA2-1R	CATTAAATTGTCACTTTTGAGGGGA
BRCA2-2F	ATGGCAGGTTGTTACGAGGC
BRCA2-2R	TGAGCTGGTCTGAATGTTCGT
BRCA2-3F	TGGCCAAAAGGAAGTCTGTT
BRCA2-3R	GTACTGGCCTGGGAACTCTC

All primers are *H. sapiens* specific. F, forward primer. R, reverse primer. The common overhangs in NGS primers are indicated by italics.

Supplemental Note. Estimation of minimum number of sequencing reads needed

a. Formula for estimation of minimum read number

We derived below formula to estimate the minimum number of amplicon NGS reads needed for a desired *P* value with a given number of experimental replicates, for a given effect size and for given knockin frequencies in a CRISPR-Select analysis (see **c**, for derivation of the formula). It should be noted that the formula serves as a guideline only, as the model incorporates certain assumptions and approximations that may not be valid for all experiments.

$$N > \left(\frac{T_s(2n-2)\sqrt{\frac{2+es^2-2es}{n}}}{es}\right)^2 \left(\frac{(1-p_1)}{p_1}+\frac{(1-p_2)}{p_2}\right)$$

, where

N: Read number

- *Total amplicon NGS reads for the given CRISPR-Select target site in the sample given as a number, e.g., 22949*

n: Experimental replicates

- *Experimental replicates given as a number, e.g., 3*

$T_s(2n-2)$:

- *To find the critical T value, consult a t-distribution table (two-tailed; e.g.* https://www.medcalc.org/manual/t-distribution-table.php) according to desired *P* value (S) and degrees of freedom, calculated by the (2n-2) formula

n: Experimental replicates

- *Experimental replicates given as a number, e.g., 3*

es: Effect size

- *The variant:WT′ ratio change given as a fraction, e.g., 0.80, meaning an 80% loss of variant alleles*

p_1: Knockin frequency of variant-of-interest

- *Given as a fraction, e.g., 0.06*

p_2: Knockin frequency of WT′.

- *Given as a fraction, e.g., 0.07*

b. Two examples of estimation of minimum read number

In below example one, a *P* value of 0.05 was desired for a CRISPR-Select experiment performed 3 times, where variant and WT′ were knocked in at 2% and 3% frequencies,

respectively and the effect size of variant was 0.2, meaning a 20% loss of variant alleles. Degrees of freedom is (2∗3-2=) 4 and with desired *P* value (S) of 0.05, *T* is 2.776 according to t-distribution table (https://www.medcalc.org/manual/t-distribution-table.php). Thereby, the minimum number of reads needed are:

$$N > \left(\frac{2.776\sqrt{\frac{2 + 0.2^2 - 2 * 0.2}{3}}}{0.2} \right)^2 \left(\frac{(1 - 0.02)}{0.02} + \frac{(1 - 0.03)}{0.03} \right) \approx 8566$$

In below example two, a *P* value of 0.05 was desired for a CRISPR-Select experiment performed 3 times, where variant and WT′ were knocked in at 2% and 3% frequencies, respectively and the effect size of variant was 0.8, meaning an 80% loss of variant alleles . Degrees of freedom is (2∗3-2=) 4 and with desired *P* value (S) of 0.05, *T* is 2.776 according to t-distribution table (https://www.medcalc.org/manual/t-distribution-table.php). Thereby, the minimum number of reads needed are:

$$N > \left(\frac{2.776\sqrt{\frac{2 + 0.8^2 - 2 * 0.8}{3}}}{0.8} \right)^2 \left(\frac{(1 - 0.02)}{0.02} + \frac{(1 - 0.03)}{0.03} \right) \approx 339$$

c. Derivation of formula for estimation of minimum read number

The formula was derived as follows: We used the ratio of variant reads to WT′ reads to test, whether the difference in ratios of two conditions (*e.g.*, two time points) is significant. The variant: WT′ ratio of the i-th sample in one condition is x_i and the variant:WT ratio of the i-th sample in another condition is y_i. Ratios are given as fractions, *e.g.*, 0.8. To test whether the means of x and y are different in *n* experimental replicates, we used equal variance t-test as follows:

$$t = \frac{\bar{x} - \bar{y}}{s_p \cdot \sqrt{\frac{2}{n}}} \quad (1)$$

, where

$$s_p = \sqrt{\frac{\sigma_x^2 + \sigma_y^2}{2}} \quad (2)$$

Effect size (*es*) is incorporated as follows:

$$\bar{y} = (1 - es)\bar{x} \quad (3)$$

Because x and y are paired, we assume that x and y have the same relative variance (α):

$$\alpha = \frac{\sigma_x}{\bar{x}} = \frac{\sigma_y}{\bar{y}} \quad (4)$$

Then,

$$\sigma_x = \bar{x}.\alpha \tag{5}$$

$$\sigma_y = \bar{y}.\alpha \tag{6}$$

$$t = \frac{es}{\alpha\sqrt{\frac{2+es^2-2es}{n}}} \tag{7}$$

The desired P value (S) for significance as well as number of experimental replicates (n) are incorporated as follows:

$$|t| > T_S(2n-2) \tag{8}$$

, which equals:

$$\left|\frac{es}{\alpha\sqrt{\frac{2+es^2-2es}{n}}}\right| > T_S(2n-2) \tag{9}$$

$$\alpha < \frac{|es|}{T_S(2n-2)\sqrt{\frac{2+es^2-2es}{n}}} \tag{10}$$

Among the total N reads of each sample, the number of m variant reads follows binomial distribution: $m \sim B(N, p_1)$, where p_1 is frequency of variant knockin given as a fraction, *e.g.*, 0.02. The number of w WT′ reads also follows binomial distribution: $w \sim B(N, p_2)$, where p_2 is frequency of WT′ knockin given as a fraction, *e.g.*, 0.02.

$$x = \frac{m}{w} \tag{11}$$

$$\sigma_m^2 = Np_1(1-p_1) \tag{12}$$

$$\sigma_w^2 = Np_2(1-p_2) \tag{13}$$

The relative variance α^2 of x is:

$$\sigma_x^2 = \sigma_{\frac{m}{w}}^2 = \sigma_m^2\left(\frac{\partial x}{\partial m}\right)_{\bar{m}}^2 + \sigma_w^2\left(\frac{\partial x}{\partial w}\right)_{\bar{w}}^2 = \frac{\sigma_m^2}{\bar{w}^2} + \frac{\sigma_w^2\bar{m}^2}{\bar{w}^4} \tag{14}$$

$$\alpha^2 = \left(\frac{\sigma_x}{\frac{\bar{m}}{\bar{w}}}\right)^2 = \left(\frac{\sigma_{\frac{m}{w}}}{\frac{\bar{m}}{\bar{w}}}\right)^2 = \left(\frac{\sigma_m}{\bar{m}}\right)^2 + \left(\frac{\sigma_w}{\bar{w}}\right)^2 \tag{15}$$

When $N \to +\infty$; $\bar{m} \to Np_1$, $\bar{w} \to Np_2$, the Eq. 15 can be written as:

$$\alpha^2 \to \frac{(1-p_1)}{Np_1} + \frac{(1-p_2)}{Np_2}, (N \to +\infty) \tag{16}$$

The Eq. 10 can be written as:

$$\frac{(1-p_1)}{Np_1} + \frac{(1-p_2)}{Np_2} < \left(\frac{es}{T_S(2n-2)\sqrt{\frac{2+es^2-2es}{n}}}\right)^2, (N \to +\infty) \tag{17}$$

$$N > \left(\frac{T_S(2n-2)\sqrt{\frac{2+es^2-2es}{n}}}{es}\right)^2 \left(\frac{(1-p_1)}{p_1} + \frac{(1-p_2)}{p_2}\right), (N \to +\infty) \tag{18}$$
