# Supplementary material for: Multiparametric and accurate functional analysis of genetic sequence variants using CRISPR-Select
Source: Nat Genet. 2022 Dec 5;54(12):1983–93. doi: 10.1038/s41588-022-01224-7 (PMC9729100; doi:10.1038/s41588-022-01224-7)
Supplement: Supplementary file 2 — Reporting Summary [file 41588_2022_1224_MOESM2_ESM.pdf]

nature portfolio

Corresponding author(s): Morten Frodin, Claus S Sørensen

Last updated by author(s): Aug 20, 2022

Reporting Summary

Nature Portfolio wishes to improve the reproducibility of the work that we publish. This form provides structure for consistency and transparency in reporting. For further information on Nature Portfolio policies, see our Editorial Policies and the Editorial Policy Checklist.

Statistics

For all statistical analyses, confirm that the following items are present in the figure legend, table legend, main text, or Methods section.

n/a	Confirmed	
☐	☒	The exact sample size (*n*) for each experimental group/condition, given as a discrete number and unit of measurement
☐	☒	A statement on whether measurements were taken from distinct samples or whether the same sample was measured repeatedly
☐	☒	The statistical test(s) used AND whether they are one- or two-sided<br>*Only common tests should be described solely by name; describe more complex techniques in the Methods section.*
☒	☐	A description of all covariates tested
☐	☒	A description of any assumptions or corrections, such as tests of normality and adjustment for multiple comparisons
☐	☒	A full description of the statistical parameters including central tendency (e.g. means) or other basic estimates (e.g. regression coefficient) AND variation (e.g. standard deviation) or associated estimates of uncertainty (e.g. confidence intervals)
☐	☒	For null hypothesis testing, the test statistic (e.g. *F*, *t*, *r*) with confidence intervals, effect sizes, degrees of freedom and *P* value noted<br>*Give P values as exact values whenever suitable.*
☒	☐	For Bayesian analysis, information on the choice of priors and Markov chain Monte Carlo settings
☒	☐	For hierarchical and complex designs, identification of the appropriate level for tests and full reporting of outcomes
☒	☐	Estimates of effect sizes (e.g. Cohen's *d*, Pearson's *r*), indicating how they were calculated

Our web collection on statistics for biologists contains articles on many of the points above.

Software and code

Policy information about availability of computer code

Data collection	Benchling; FACSChorus software (version 2.0).
Data analysis	CRISPResso2 (version 2.1.0); FlowJo (version 10.4); ICE-Analysis online tool (version 3.0); GraphPad Prism (version 9.2.0).

For manuscripts utilizing custom algorithms or software that are central to the research but not yet described in published literature, software must be made available to editors and reviewers. We strongly encourage code deposition in a community repository (e.g. GitHub). See the Nature Portfolio guidelines for submitting code & software for further information.

Data

Policy information about availability of data

All manuscripts must include a data availability statement. This statement should provide the following information, where applicable:

- Accession codes, unique identifiers, or web links for publicly available datasets
- A description of any restrictions on data availability
- For clinical datasets or third party data, please ensure that the statement adheres to our policy

All data sets are available within the article, or from the corresponding authors upon request. The sequencing data have been deposited to the NCBI Sequence Read Archive database with the accession number PRJNA759404. The plasmid harboring inducible Cas9 which was used to make the iCas9-MCF10A cell pool is available from Addgene (plasmid #170805). Mutation data on BRCA2 variants from ClinVar were accessed through https://www.ncbi.nlm.nih.gov/clinvar/. For CRISPR-Select cassette design, the Human Splicing Finder online tool was accessed through http://www.umd.be/HSF3/ and the Codon Usage Database was accessed through http://www.kazusa.or.jp/codon.

Field-specific reporting

Please select the one below that is the best fit for your research. If you are not sure, read the appropriate sections before making your selection.

☒ Life sciences ☐ Behavioural & social sciences ☐ Ecological, evolutionary & environmental sciences

For a reference copy of the document with all sections, see nature.com/documents/nr-reporting-summary-flat.pdf

Life sciences study design

All studies must disclose on these points even when the disclosure is negative.

Sample size	Sample-size calculations were not performed. We chose a sample size that ensured that consistent and significant results were obtained between replicates (n=3 or n=4). For all NGS analysis, 100 ng genomic DNA were used as template, except for the apoptosis and γ-H2AX assays that used 50 ng, to ensure a representative sample size for knock-in determinations for each experiment.
Data exclusions	No data were excluded.
Replication	All experiments were repeated three times or, for in vivo experiments, 3 or 4 mice were included per experimental group. All attempts at replication were successful.
Randomization	Allocation of samples into experimental groups was performed randomly and as stated in the Methods. For in vitro experiments involving inhibitor treatment, samples were randomly allocated to vehicle- or inhibitor-treated groups. For in vivo inhibitor studies, mice were randomly distributed into vehicle-or inhibitor-treated groups. Except for expert panel variants, the significance of BRCA2 variants in breast cancer was determined without prior knowledge of the pathogenicity status of the variants, which was then assessed with CRISPR-Select.
Blinding	Cell culture and FACS experiments were set by an researcher and subsequent NGS or Sanger sequencing experiments and data analysis were performed blindly by another researcher. Tumor measurements of vehicle- or inhibitor-treated mice were also performed blindly as was subsequent NGS sequencing and data analysis of the tumor samples.

Reporting for specific materials, systems and methods

We require information from authors about some types of materials, experimental systems and methods used in many studies. Here, indicate whether each material, system or method listed is relevant to your study. If you are not sure if a list item applies to your research, read the appropriate section before selecting a response.

Materials & experimental systems

n/a	Involved in the study	
☐	☒	Antibodies
☐	☒	Eukaryotic cell lines
☒	☐	Palaeontology and archaeology
☐	☒	Animals and other organisms
☐	☒	Human research participants
☒	☐	Clinical data
☒	☐	Dual use research of concern

Methods

n/a	Involved in the study	
☒	☐	ChIP-seq
☐	☒	Flow cytometry
☒	☐	MRI-based neuroimaging

Antibodies

Antibodies used	S-phase FACS detection: Alexa Fluor® 488 azide (Thermo Fisher Scientific, #C10425, lot number 2161907, this antibody is part of a kit and its concentration is proprietary information); Apoptosis FACS detection: Alexa Fluor™ 488 Anti-BrdU (Thermo Fisher Scientific, #A23210, lot number 2156495, 1:20 dilution); DNA damage assay: Anti-phospho-Histone H2A.X (Ser139) Antibody, clone JBW301 (Millipore, #05-636, lot number 3292608, 1:1000 dilution), Goat anti-Mouse IgG (H+L) Cross-Adsorbed Secondary Antibody, Alexa Fluor 488 (Thermo Fisher Scientific, #A-11001, lot number 2247988, 1:1000 dilution).
Validation	Validation of antibodies for the intended used and species was available on the website of the manufacturer:<br>Anti-phospho-Histone H2A.X (Ser139) Antibody, clone JBW301: this antibody detected pSer259 histone H2A.X in acid extracted histone lysates from Jurkat cells treated with staurosporine;<br>Alexa Fluor® 488 azide, Alexa Fluor™ 488 Anti-BrdU : the antibodies were tested in a typical application and in accordance with the product information sheet;

Eukaryotic cell lines

Policy information about cell lines

Cell line source(s)	MCF10A, H358, Hep3B, MCF7 and HEK 293T cells were purchased from American Type Culture Collection (ATCC).The iCas9-MCF10A clonal cell line with Cas9 expressed from stably integrated TRE3G Edit-R Inducible Lentiviral Cas9 construct was a gift from Roderick L. Beijersbergen, The Netherlands Cancer Institute, and originally purchased from ATCC. Human colon organoids were generated from biopsy samples of human colon tissue taken from a healthy donor at Herlev Hospital, Denmark.
Authentication	Human colon organoids were not authenticated. The iCas9-MCF10A clonal cell line gifted by Roderick L. Beijersbergen was originally purchased from ATCC and authenticated by the supplier. All the remaining cell lines mentioned above were purchased from ATCC and authenticated by the supplier. Authentication data available from ATCC included STR profiling and cell line morphology.
Mycoplasma contamination	All cell lines tested negative for mycoplasma contamination.
Commonly misidentified lines (See ICLAC register)	No commonly misidentified cell lines were used.

Animals and other organisms

Policy information about studies involving animals; ARRIVE guidelines recommended for reporting animal research

Laboratory animals	4-5-week-old athymic female mice (Charles River Laboratories, strain code 490).
Wild animals	This study did not involve wild animals.
Field-collected samples	This study did not involve samples collected from the field.
Ethics oversight	The experiments were approved by the Danish Experimental Inspectorate (License 2019-15-0201-00307).

Note that full information on the approval of the study protocol must also be provided in the manuscript.

Human research participants

Policy information about studies involving human research participants

Population characteristics	Human colon organoids were established from biopsy samples of human colon tissue from a healthy woman, aged 54, at Herlev Hospital.
Recruitment	Human organoids from a healthy control were isolated from up to six sigmoid colon biopsy specimens obtained during a routine colonoscopy at Department of Gastroenterology, Herlev Hospital, University of Copenhagen, performed due to gastrointestinal symptoms, but in whom all clinical investigations subsequently turned out to be normal, e.g., irritable bowel syndrome. Moreover, the healthy control was a subject without any known diseases and free of daily medication. Lactating or pregnant subjects as well as subjects with psychiatric or neurological disorders that would affect decision-making were excluded from participating. No biases were present when recruiting the patient.
Ethics oversight	This study was approved by the Scientific Ethics Committee of the Copenhagen Capital Region. All patients provided written informed consent and the study was performed in accordance with ethical guidelines (Protocol No. H-18005342).

Note that full information on the approval of the study protocol must also be provided in the manuscript.

Flow Cytometry

Plots

Confirm that:

- [x] The axis labels state the marker and fluorochrome used (e.g. CD4-FITC).
- [x] The axis scales are clearly visible. Include numbers along axes only for bottom left plot of group (a 'group' is an analysis of identical markers).
- [x] All plots are contour plots with outliers or pseudocolor plots.
- [x] A numerical value for number of cells or percentage (with statistics) is provided.

Methodology

Sample preparation	For proliferation or apoptosis staining of MCF10A cells, after CRISPR-Select editing with the PIK3CA H1047R cassette, cells were incubated in culture medium without serum or any supplements for five days. For proliferation staining of H358 cells, after CRISPR-Select editing with the KRAS G12C cassette, cells were treated with 0.1 % DMSO or 0.12 % AMG 510 for three

days. For the DNA damage assay of MCF10A cells, after CRISPR-Select editing with the BRCA2 T2722R cassette, cells were grown in complete medium for four days. For all experiments, prior to staining, cells were dissociated using trypsin.

Instrument	BD FACSMelody (BD Bioscience).
Software	FACSChorus Software; FlowJo.
Cell population abundance	For detection of S-phase and apoptosis markers, cells were sorted into positive and negative populations. For the DNA damage assay of MCF10A cells, cells were sorted into high and low populations based on their fluorescent level of Alexa Fluor 488-bound γH2AX. Stringent gating was always used to assure sufficient separation of the sorted populations (high/low and positive/negative).
Gating strategy	For all flow cytometry experiments, cells were initially gated for live cells using SSC-A/FSC-A (gate A). Next, singlets were sorted for by gating for SSC-W/SSC-H (gate B) and FSC-W/FSC-H (gate C). As final gating, S-phase positive and negative cells (proliferation assays), apoptotic and non-apoptotic cells (apoptosis assays) and γH2AX-high and -low cells (DNA damage marker) were sorted for using Alexa Fluor 488/PI as flurophores (gate D), given that positive and γH2AX-high populations were Alexa Fluor 488-positive. The boundaries for the sorted cell populations were defined based on comparison with negative control samples.

☐ Tick this box to confirm that a figure exemplifying the gating strategy is provided in the Supplementary Information.
